# Perennial malaria chemoprevention with and without malaria vaccination to reduce malaria burden in young children: a modelling analysis

**DOI:** 10.1186/s12936-023-04564-9

**Published:** 2023-04-24

**Authors:** Manuela Runge, Anne Stahlfeld, Monique Ambrose, Kok Ben Toh, Semiu Rahman, Omowunmi F. Omoniwa, Caitlin A. Bever, Olusola Oresanya, Perpetua Uhomoibhi, Beatriz Galatas, James K. Tibenderana, Jaline Gerardin

**Affiliations:** 1 Department of Preventive Medicine, Institute for Global Health, Northwestern University, Chicago, IL USA; 2 Institute for Disease Modeling, Bill and Melinda Gates Foundation, Seattle, USA; 3 Malaria Consortium Nigeria, 33 Pope John Paul Street, Off Gana Street, Maitama, Abuja-FCT Nigeria; 4 Global Malaria Programme, World Health Organization, Geneva, Switzerland; 5 National Malaria Elimination Programme, Federal Ministry of Health, Abuja, Nigeria; 6 Malaria Consortium Headquarters, 244-254 Cambridge Heath Rd, E2 9DA London, UK

**Keywords:** PMC, RTS,S, Nigeria, Mathematical modeling, Malaria modeling, Malaria prevention, Malaria vaccine, Malaria chemoprevention, EMOD

## Abstract

**Background:**

A recent WHO recommendation for perennial malaria chemoprevention (PMC) encourages countries to adapt dose timing and number to local conditions. However, knowledge gaps on the epidemiological impact of PMC and possible combination with the malaria vaccine RTS,S hinder informed policy decisions in countries where malaria burden in young children remains high.

**Methods:**

The EMOD malaria model was used to predict the impact of PMC with and without RTS,S on clinical and severe malaria cases in children under the age of two years (U2). PMC and RTS,S effect sizes were fit to trial data. PMC was simulated with three to seven doses (PMC-3-7) before the age of eighteen months and RTS,S with three doses, shown to be effective at nine months. Simulations were run for transmission intensities of one to 128 infectious bites per person per year, corresponding to incidences of < 1 to 5500 cases per 1000 population U2. Intervention coverage was either set to 80% or based on 2018 household survey data for Southern Nigeria as a sample use case. The protective efficacy (PE) for clinical and severe cases in children U2 was calculated in comparison to no PMC and no RTS,S.

**Results:**

The projected impact of PMC or RTS,S was greater at moderate to high transmission than at low or very high transmission. Across the simulated transmission levels, PE estimates of PMC-3 at 80% coverage ranged from 5.7 to 8.8% for clinical, and from 6.1 to 13.6% for severe malaria (PE of RTS,S 10–32% and 24.6–27.5% for clinical and severe malaria, respectively. In children U2, PMC with seven doses nearly averted as many cases as RTS,S, while the combination of both was more impactful than either intervention alone. When operational coverage, as seen in Southern Nigeria, increased to a hypothetical target of 80%, cases were reduced beyond the relative increase in coverage.

**Conclusions:**

PMC can substantially reduce clinical and severe cases in the first two years of life in areas with high malaria burden and perennial transmission. A better understanding of the malaria risk profile by age in early childhood and on feasible coverage by age, is needed for selecting an appropriate PMC schedule in a given setting.

**Supplementary Information:**

The online version contains supplementary material available at 10.1186/s12936-023-04564-9.

## Background

Malaria burden in sub-Saharan Africa remains intolerably high despite the availability of a range of preventive and therapeutic malaria interventions. In 2021, approximately 247 million cases and 619 thousand deaths due to malaria occurred globally, most of them in children under the age of 5 years [1]. While insecticide-treated bed nets and prompt and effective case management have been the cornerstone of malaria control over the last two decades, these interventions alone have not been sufficient to address the malaria burden [2]. Seasonal malaria chemoprevention (SMC) is recommended in many regions with highly seasonal transmission, and 45 million children under five years of age received SMC in 2021 [1]. However, in perennial transmission areas, policy adoption of malaria chemoprevention in children has been limited to a single country [3], leaving many children unprotected during their first few years of life.

Perennial malaria chemoprevention (PMC) is the administration of antimalarials to children at the highest risk of malaria at specific ages throughout the year [4]. The intervention aims to provide protection from malaria disease while allowing for some acquisition of natural immunity [5]. A pooled analysis of clinical trials conducted in the early 2000s reported a 22–30% reduction in clinical episodes in infants due to malaria chemoprevention with three to four doses [6, 7]. In 2010, the World Health Organization (WHO) recommended three doses of sulfadoxine-pyrimethamine (SP) administered at ten weeks, fourteen weeks, and nine months of age, and referred to the intervention as intermittent preventive treatment in infants (IPTi) [8].

Despite initial recommendation in 2010, IPTi has been programmatically implemented only in Sierra Leone [3], and urgent action has been called for to encourage its adoption in more countries’ malaria planning [9]. In mid-2022, the WHO updated its guidelines for IPTi, removing the fixed number of doses at specific ages and relabeled the intervention as PMC [4]. These changes allow for flexible targeting of doses to children most vulnerable to severe malaria and death and encourage countries to tailor implementation of the intervention based on local contextual factors. To generate stronger evidence of the impact of PMC, especially for subnational tailoring, new data for different deployment schedules in current epidemiological and operational contexts are required. Several PMC field studies are currently in planning or ongoing in both East- and West-African perennial settings to assess the impact of three or more doses [10, 11].

Chemoprevention is not the only option for pharmaceutical malaria prevention in young children. In October 2021, the WHO recommended the first malaria vaccine RTS,S/AS01 as part of a comprehensive package of malaria control [12]. RTS,S efficacy among children receiving four doses between five and seventeen months of age followed up for 48 months was 39% against clinical and 29% against severe malaria [13]. The idea of combining chemoprevention with malaria vaccination to achieve greater impact than with either intervention alone is not new [14], and was tested with SMC and RTS,S in Burkina Faso and Mali [15]. That study showed a marked joint impact of RTS,S and SMC and non-inferiority of RTS,S to SMC [15]. However, the combination of PMC with a malaria vaccine has not yet been studied although both interventions share the same delivery platform through the Expanded Programme for Childhood Immunization (EPI).

The new flexibility in scheduling and timing of PMC and its possible combination with a vaccine, that is expected to be in high demand, are key considerations for the strategic planning of these interventions in malaria endemic countries. Additionally, constantly progressing epidemiological changes, complex within-host and immunity dynamics, supply and operational constraints as well as different sociocultural contexts complicate determining the accurate public health impact in countries. Mathematical modelling has been widely used to assess the potential impact of malaria interventions such as RTS,S [16], drug-based interventions [17–19], or vector control [20, 21], and to identify knowledge gaps, key assumptions, or concepts to test.

This modelling study, investigated the technically feasible impact of PMC on malaria burden during early childhood. Various PMC schedules with up to seven doses of SP were considered and simulated alone or in combination with the malaria vaccine RTS,S at its recommended schedule. Model outcomes were used to describe generalizable trends in intervention impact by age across a wide range of transmission levels at a fixed target coverage. In addition, reported EPI coverage levels in Southern Nigeria were used to estimate the potential operational impact. Nigeria presents a relevant setting to explore the impact of large-scale PMC implementation as it continues to record the highest malaria burden globally [1], and the introduction of PMC in the Southern area which experiences perennial transmission is important to save lives.

## Methods

### Mathematical model

Simulations were run using the individual-based malaria transmission model EMOD [22, 23], version 2.20 [24]. The model can incorporate intervention campaigns targeted to specific age ranges or times of the year and has previously been used to simulate chemoprevention [25, 26] and vaccines [27]. EMOD includes age-dependent transmission risk, acquisition of partial immunity based on cumulative exposure [28], and maternal antibody protection against malaria disease in infants three to six months of age [29]. Modelled severe incidence was previously calibrated to data from five sites in The Gambia and Kenya [29, 30]. In the simulation model, uncomplicated and severe malaria cases are treated with artemether-lumefantrine using drug parameters previously calibrated for EMOD [25].Simulation inputs and outputs were processed using Python 3.6 [31], and calculation of outcome measures and their visualization in R 4.0.2 [32].

### PMC parameterization

**Fig. 1:**
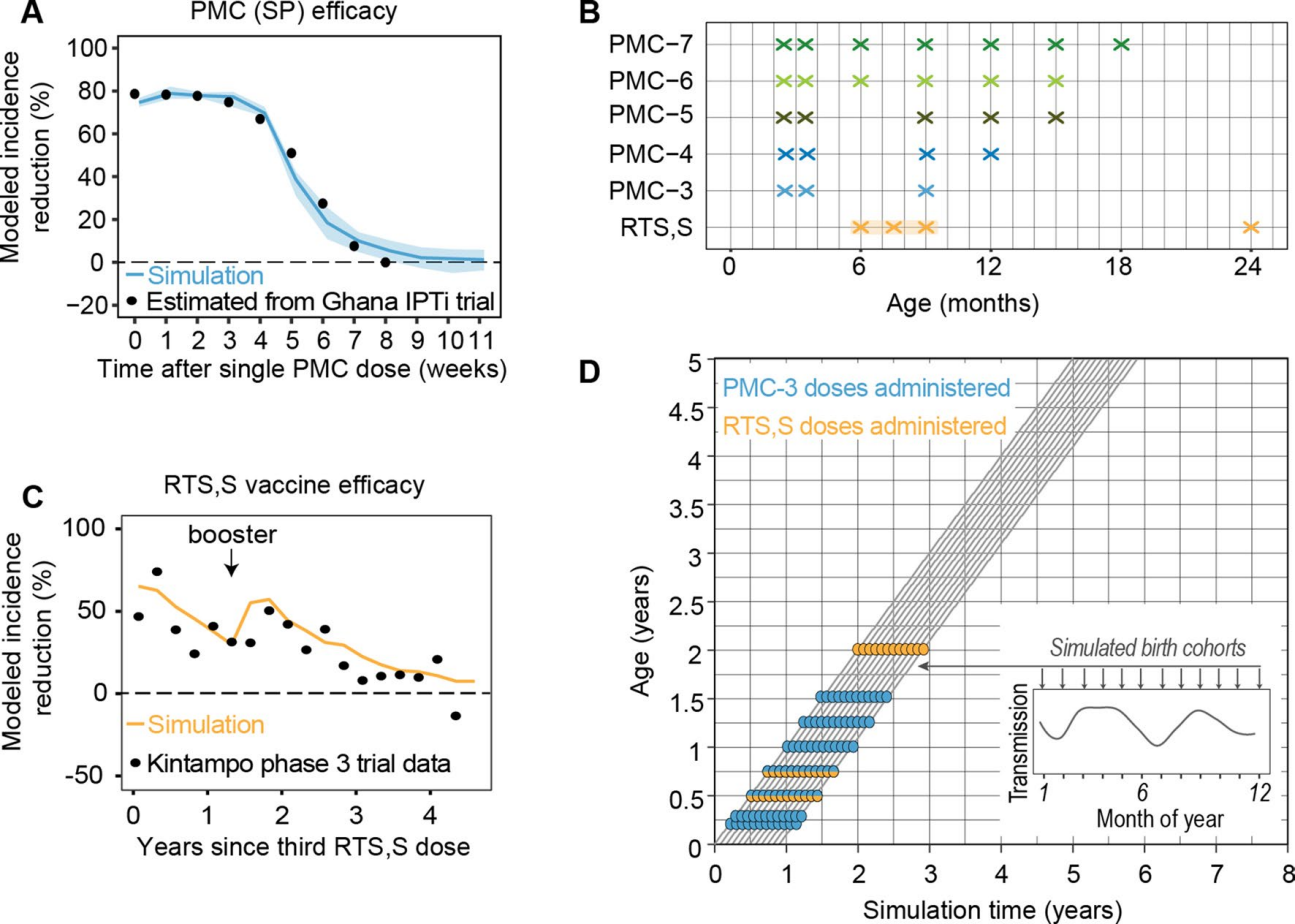
Modelled intervention efficacies, intervention schedules, and simulated cohort populations. **A** Intervention efficacy of modelled SP. Simulations ran with an EIR of 32 infectious bites per person per annum (ibpa), 60% effective clinical treatment coverage, and 95% coverage of a single dose of PMC with SP. Reference points are smoothed estimates based on the averaged effect of four doses in children less than 15 months in Ghana 2005 trial [33, 34]. **B** Age schedules of PMC and RTS,S deployments. **C** Intervention efficacy of the modelled malaria vaccine RTS,S [29] at 100% coverage. Simulations ran with an EIR of 11 ibpa and 90% treatment coverage of clinical cases. Data points correspond to RTS,S Phase 3 trial data from the Kintampo trial site [13], obtained from [27]. **D** Schematic of birth cohorts (n = 12) in simulation setup, truncated at five of ten follow-up years. Colored points indicate events when individuals receive either PMC, RTS,S, or both interventions. The inset figure shows the transmission seasonality relative to birth month of each cohort

PMC was simulated with a prophylactic effect that prevents the user from new infections. The effect parameters were fitted to the estimated efficacy curve of a single dose of SP on clinical cases based on a randomized controlled trial conducted in Ghana in the early 2000s [33, 34]. Because the simulated PMC does not remove existing infections after administration, the maximum efficacy of a single dose is only reached after seventeen days, with a steep increase after ten days. To better fit the trial data, a ten-day offset to the scheduled PMC doses was included (Additional file 1: Fig. A1.1.0). The modelled PMC efficacy was held constant for fifteen days after it reached its maximum, followed by an exponential decay (half-life of twenty days), fully losing protection at eight weeks at moderate transmission (Fig. 1A). The initial efficacy among simulated individuals was varied following a normal distribution with mean efficacy of 0.8 and a standard deviation of 0.025, truncated at 0.75 and 0.9. Two doses given within a 28-day window had an additive effect with maximum efficacy of 1 and prolonged effect duration. A simulation matched to the study site in Ghana with four doses of SP showed a slightly lower impact in infants than what was observed, likely due to the very high transmission intensity (reported annual EIR = 418) at the time of the study [33], whereas overall pooled effect sizes were well reproduced (see Additional file 1: A1.1).

The modelled 3-dose PMC schedule (PMC-3) was based on the 2010 WHO recommendation [8] with doses administered at the second and third diphtheria-tetanus-pertussis (DTP) vaccinations at ten and fourteen weeks of age, and at measles vaccination at nine months of age. Other PMC schedules with up to seven doses before 18 months of age were selected based on discussions with operational researchers and other schedules suggested in literature [14] (Fig. 1B).

In the simulation, PMC was administered regardless of malaria infection or treatment status of individuals, and each dose was distributed independently of whether the child had received a previous dose. Children received each PMC dose exactly as scheduled without delays. PMC coverage per dose was set to 80%, corresponding to the district-level minimum WHO vaccination target by 2020 [35]; or informed by State-level EPI coverage in Southern Nigeria. In addition, coverage levels between zero and 100% in intervals of 20% were explored (see Additional file 1).

### Malaria vaccine (RTS,S) parameterization

To simulate a malaria vaccine, a previously established parameterization of RTS,S that was fitted to Phase-3 trial data [13, 27] was used. The simulated RTS,S schedule followed WHO recommendations with a three-dose primary series at six, seven and nine months, and one booster at 24 months of age [4]. In the model, it was assumed that the impact of the first three doses takes effect only after the third dose (Fig. 1C). We defined RTS,S vaccination coverage was defined as the fraction of children nine months of age who received all three priming doses and fixed the booster coverage at 80% of those who had received all three priming doses. In the model, children who did not receive all three priming doses were not eligible for a booster and among individual children, coverage with RTS,S was independent of coverage with PMC.

### Simulated malaria transmission and seasonality

Simulations were run with forced levels of transmission (fixed mosquito to human transmission as the impact of PMC and RTS,S on community transmission is expected to be minimal. The transmission levels varied across months within a year but not from year to year. Monthly transmission followed a perennial pattern based on modelled monthly entomological inoculation rates (EIR) from a previous EMOD modelling analysis for Nigeria, that calibrated modelled incidence to monthly case data [36]. The selected transmission levels ranged from one to 128 infectious bites per person per year (ibpa) to obtain different incidence curves by age for clinical and severe malaria as they might occur across sub-Saharan Africa. The corresponding simulated prevalence according to rapid diagnostic test ranged from < 1 to 75% (*Pf*PR_U5_ <1 to 80%) in children U2, the clinical incidence from 135 to 5,500, and severe incidence from < 1 to 97 per 1000 population U2 (see Additional file 1: Fig. A1.2.2). It should be noted that the simulated incidence levels reflect a health care seeking rate and reporting rate of 100% and are not directly relatable to country reported incidences. In the analysis, the simulated transmission levels were referred to low (EIR = 1,4 with *Pf*PR_U5_ 3–11%), moderate (EIR = 8, 16 with *Pf*PR_U5_ 21–36%), high (EIR = 32 with *Pf*PR_U5_ ~55%), and very high transmission (EIR = 64, 128 with *Pf*PR_U5_ 70–80%). These are specific to these simulated transmission levels and differ from the endemicity classes defined by the WHO that uses prevalence cutoff of 1%, 10%, 35% for very low, low, moderate, and high endemicity [37].

### Simulation setup and scenarios

The simulation ran for a closed birth cohort of 30,000 individuals that were followed up for ten years in batches of twelve cohorts, one for each month (Fig. 1D). Two sets of input parameters were used: (1) geographic-agnostic inputs to explore relationships and intervention effect sizes across a wide range of transmission levels, and (2) geographic-specific inputs (transmission, seasonality, treatment coverage, PMC and RTS,S coverage) to approximate operational intervention impact in Southern Nigeria. The input parameters and scenarios for both setups are presented in Table 1, and further details are available in the Supplement.

**Table 1:** Model parameters and scenarios

Parameter	Description/unit	Geographic-agnostic	Geographic-specific
Geography	Malaria setting simulation corresponds to	Not specified	20 States in Southern Nigeria
Population	Simulated total population	30,000 (closed)	30,000 (closed)
Birth cohorts	Number of population cohorts simulated from birth	12	12
Stochastic realizations	Number of random seeds for each scenario and birth cohort	5	5
Transmission intensity	annual EIR in infectious bites per person per annum (ibpa)	1, 4, 8, 16, 32, 64, 128	Derived from simulated relationship between EIR and *Pf*PR U5; EIR then estimated from *Pf*PR U5 by rapid diagnostic test per State from NDHS 2018 [38])
Transmission seasonality	Monthly EIR	Derived from [39] (details in Additional file 1: A1.2)	Taken from [36], aggregated per State.
Clinical treatment coverage	% of clinical episodes effectively treated	60	Obtained from NDHS 2018 for each State [38]
Severe treatment coverage	% of severe episodes effectively treated	80	80
PMC coverage	% of population U2 receiving PMC doses at target ages per dose at random	80	Target: 80, operational: informed by EPI coverage in Southern Nigeria for each State (NDHS 2018 [38])
PMC dosing schedule	Months of age at which child receives PMC	PMC-3: 2.5, 3.5, 9 PMC-4: 2.5, 3.5, 9, 12 PMC-5: 2.5, 3.5, 9, 12, 15 PMC-6: 2.5, 3.5, 6, 9, 12, 15 PMC-7: 2.5, 3.5, 6, 9, 12, 15, 18	PMC-3: 2.5, 3.5, 9 PMC-5: 2.5, 3.5, 9, 12, 15 PMC-7: 2.5, 3.5, 6, 9, 12, 15, 18
RTS,S coverage primary sequence	% of children receiving all 3 primary doses (combined at 9 months of age)	80	target: 0.8, operational: informed by EPI coverage for each State (NDHS 2018 [38])
RTS,S booster coverage	% of children receiving booster out of those received full primary sequence	80	80
RTS,S schedule	Months of age at which child receives RTS,S dose	6, 7.5, 9 (initial 3-dose priming sequence) + 24 (booster)	6, 7.5, 9 (initial 3-dose priming sequence) + 24 (booster)

*NDHS* Nigeria Demographic Health Survey, *EIR* entomological inoculation rate, *PMC* perennial malaria chemoprevention, *Ux* under the age of x years, *Pf*PR *Plasmodium falciparum* parasite rate

#### Analysis of model outcomes

Simulation outputs were analysed by age in weeks or aggregated into age groups with a minimum age of three months and maximum ages of one, two, or five years (U1, U2, and U5 groups respectively). Predictions across the twelve birth cohorts (one for each month) were used to calculate the mean and 90% prediction intervals (90% PI). Intervention impact was described using clinical and severe cases averted per 1000 population per year, or relative reduction in clinical and severe cases, also referred to as protective efficacy (PE), using the scenario without zero PMC and zero RTS,S coverage as counterfactual.

#### Sensitivity analyses

Additional simulations were run to assess parameters of uncertainty related to maternal antibody protection, treatment rate for clinical cases, and age-varying treatment rate (see Additional file 1: A1.3).

### Application to Southern Nigeria

To approximate the potential operational impact of PMC and RTS,S, a model framework parameterized with data specific to Southern Nigeria corresponding to the situation in 2018 before COVID-19 was used. Several indicators were extracted from the Nigeria Demographic Health Survey 2018 (NDHS) [38], including state-level malaria prevalence in children U5 (*Pf*PR_U5_) based on rapid diagnostic tests, EPI coverage in children 12–23 months of age, and case management coverage in children U5 using the *rdhs* package in R [39], The *Pf*PR_U5_ -EIR relationship from the geographic-agnostic simulation runs was used to obtain appropriate input EIR level for each State. Transmission seasonality multipliers were obtained from a previous modelling analysis in Nigeria using EMOD [36] (see Additional file 1: Fig A1.4.3).

In the analysis two coverage scenarios were considered: a target coverage of 80% and an operational coverage scenario using adjusted EPI coverages. The State-level EPI coverage levels (using DTP-2, DTP-3, and measles vaccination touchpoints) in NDHS 2018 were downscaled based on observed differences between vaccination and IPTi coverage during implementation in Sierra Leone [3] (ratios of 0.83, 0.95 and 0.69 for each of the three EPI touchpoints). For the PMC dose at six months of age, the average coverage of the prior and next vaccination points was taken, and for any PMC doses after nine months of age the same coverage as at the 9-month vaccination point was used.

Simulation results from the geographic-specific model were generated for each of 20 States in Southern Nigeria and, using the mean of five stochastic runs, aggregated to all of Southern Nigeria using the population-weighted mean for rates and sum for count outcomes. Population-weighted standard deviations were used to calculate confidence intervals. Clinical and severe cases as well as relative reductions in cases per 1000 population per year were calculated for each age group. Modelled malaria case estimates include all symptomatic cases and not only those that seek care and are captured by surveillance systems. The model outputs were used to calculate annual cases and cases averted for the total population under the age of two years in Southern Nigeria by rescaling the simulated population to match Nigeria population data. Total population estimates for Southern Nigeria were obtained from GeoPode Version 2 for 2019 [40], and multiplied by 6.8% to approximate the share of the population under the age of two years (further details described in Additional file 1: A1.4).

## Results

### Projected malaria incidence by age and transmission intensity under PMC and/or RTS,S

**Fig. 2:**
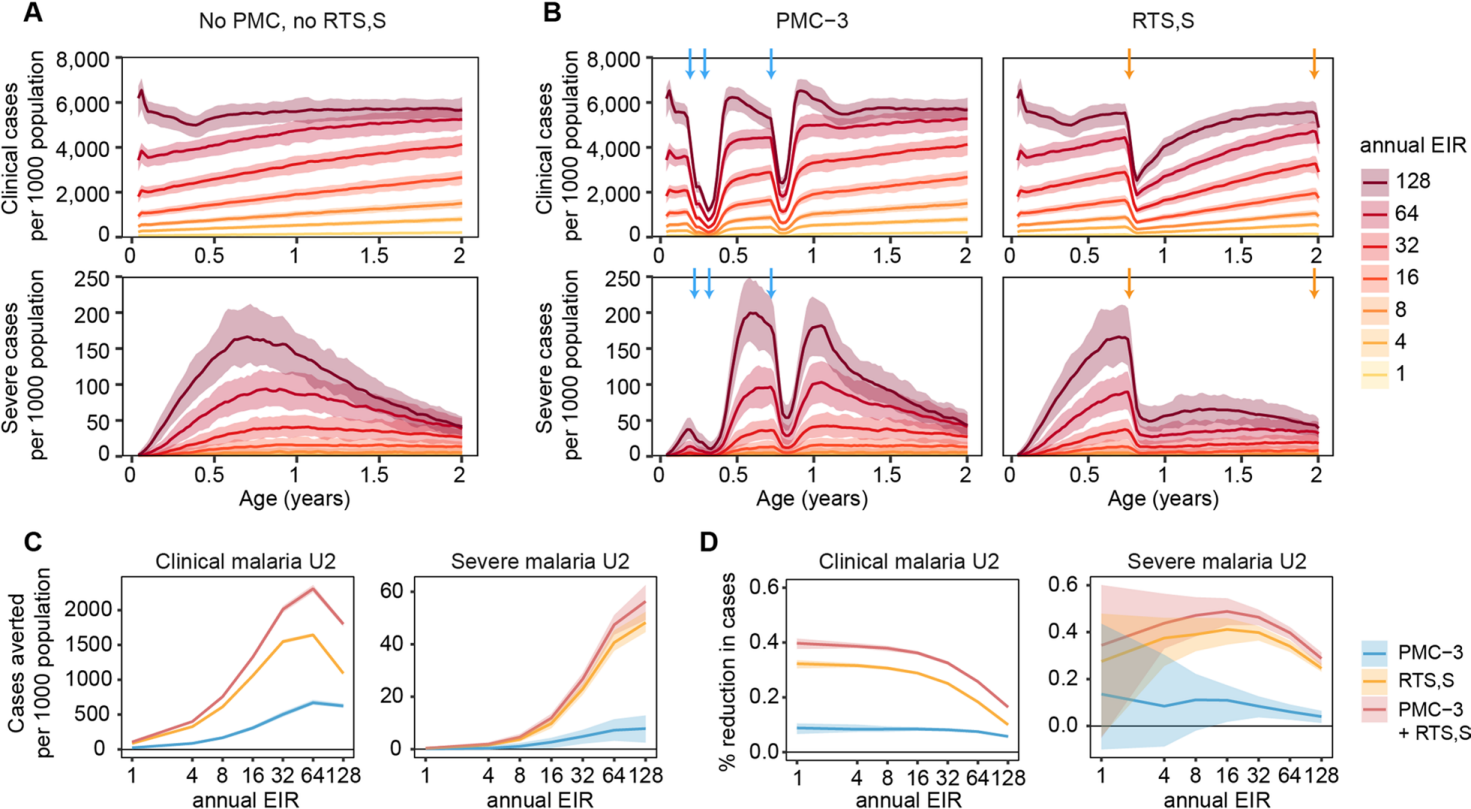
Projected clinical and severe malaria cases and cases averted by PMC-3 and/or RTS,S. **A** Clinical and severe malaria cases per 1000 population per year by age without either RTS,S or PMC-3. Projections were smoothed using a 3-week rolling average. **B** Clinical and severe malaria cases per 1000 population per year by age with PMC-3 or RTS,S at 80% coverage. The arrows indicate the timing of each PMC dose, or the 3rd RTS,S priming dose plus booster dose. **C** Clinical and severe cases averted per 1000 population per year in children U2 by transmission intensity with PMC-3, RTS,S, or both PMC-3 and RTS,S. **D** Percent reduction in clinical and severe cases in children U2 by transmission intensity and intervention. **A**–**D** The lines show the average across birth cohorts and stochastic replications, and the shaded areas the 90% prediction interval

To generate varying age-incidence curves, simulations were run in a geography-agnostic setup under annual EIRs ranging from one to 128 ibpa with fixed clinical treatment coverage of 60%. In the absence of PMC or RTS,S, clinical malaria cases peaked at around two years of age at the highest simulated transmission intensity and shifted to older ages for lower transmission intensities. Severe malaria cases were highest between six months to one year of age across the simulated transmission levels and decreased to low numbers by the end of 2 years of age, and to very low numbers (< 1 case per 1000 population U2) by the end of 3 years (Fig. 2A, Additional file 1: Fig A1.2.3 A).

The overall impact of PMC-3 (PMC with 3 doses, Fig. 1B) was greatest among children under the age of one year, and the impact of RTS,S after the first year of life, until four to five years of age (Fig. 2B, Additional file 1: Fig A1.2.3B). Under PMC-3 at 80% coverage per dose, cases dropped by around 60% after every dose for one month before resurging to pre-dose levels. PMC-3 only averted cases in children U1 but not beyond, since the expected duration of effect of the last PMC-3 dose at nine months wanes before the child reaches one year of age. Under RTS,S at 80% coverage, and 80% coverage with the booster dose among those who received the primary series, cases were reduced by around 60% after the third priming dose and by 50% after the booster dose at high transmission. Protection after the priming series and booster lasted from several months to a few months depending on transmission intensity (Additional file 1: Fig A1.1.4).

For each of the scenarios, the cumulative number of cases averted in children U2 increased with increasing transmission intensity (EIR) until reaching highest level of simulated transmission of 128 ibpa (clinical incidence of 5500 per 1000 population U2, PfPR_U2_ 75%), before dropping for clinical cases, whereas for severe cases continued increasing, although at lower rate (Fig. 2C). The trends in impact by transmission were different for impact measures on relative scale, with the PE in clinical cases remaining constant before gradually decreasing after transmission intensity reached 16 ibpa (clinical incidence of 2000 per 1000 population U2). Whereas the PE in severe cases increased gradually by transmission, before dropping after transmission intensity reached 32 ibpa (clinical incidence of 3000 per 1000 population U2 (Fig. 2D). These trends were more pronounced for RTS,S and the combination of both interventions but less so for PMC-3 alone which had a smaller impact (PE_clinicalU2_: PMC-3 5.7–8.8%; RTS,S 10–32%; PE_severeU2_: PMC-3 6.1–13.6%; RTS,S 24.6–27.5%).

Under the PMC-3 scenario in combination with RTS,S, a larger impact than for either intervention alone was projected, with a greater additional impact by RTS,S than by PMC-3 (Fig. 2C, D). For instance, compared to PMC-3 alone, the combination averted 2.8–4.5 times more clinical and 3.2–7.1 times more severe cases in children U2 on average across the simulated transmission levels. Whereas PMC-3 in combination with RTS,S compared to RTS,S alone averted 1.2–1.7 and 1.2–1.3 times more cases in children U2 for clinical and severe malaria, respectively.

### Influence of age at first PMC dose in a PMC-3 schedule

PMC was initially recommended with three doses given at 2.5, 3.5, and nine months of age [8]. At the time of the first dose, at 2.5 months of age, children might still be protected against severe disease by maternal antibodies [43], and the next dose closely follows at 3.5 months of age. Therefore, the single contribution of the 2.5-month dose to the overall PMC-3 impact in children U2 was assessed and the impact of changing its delivery to instead occur at 6, 12, or 15 months of age evaluated.

**Fig. 3:**
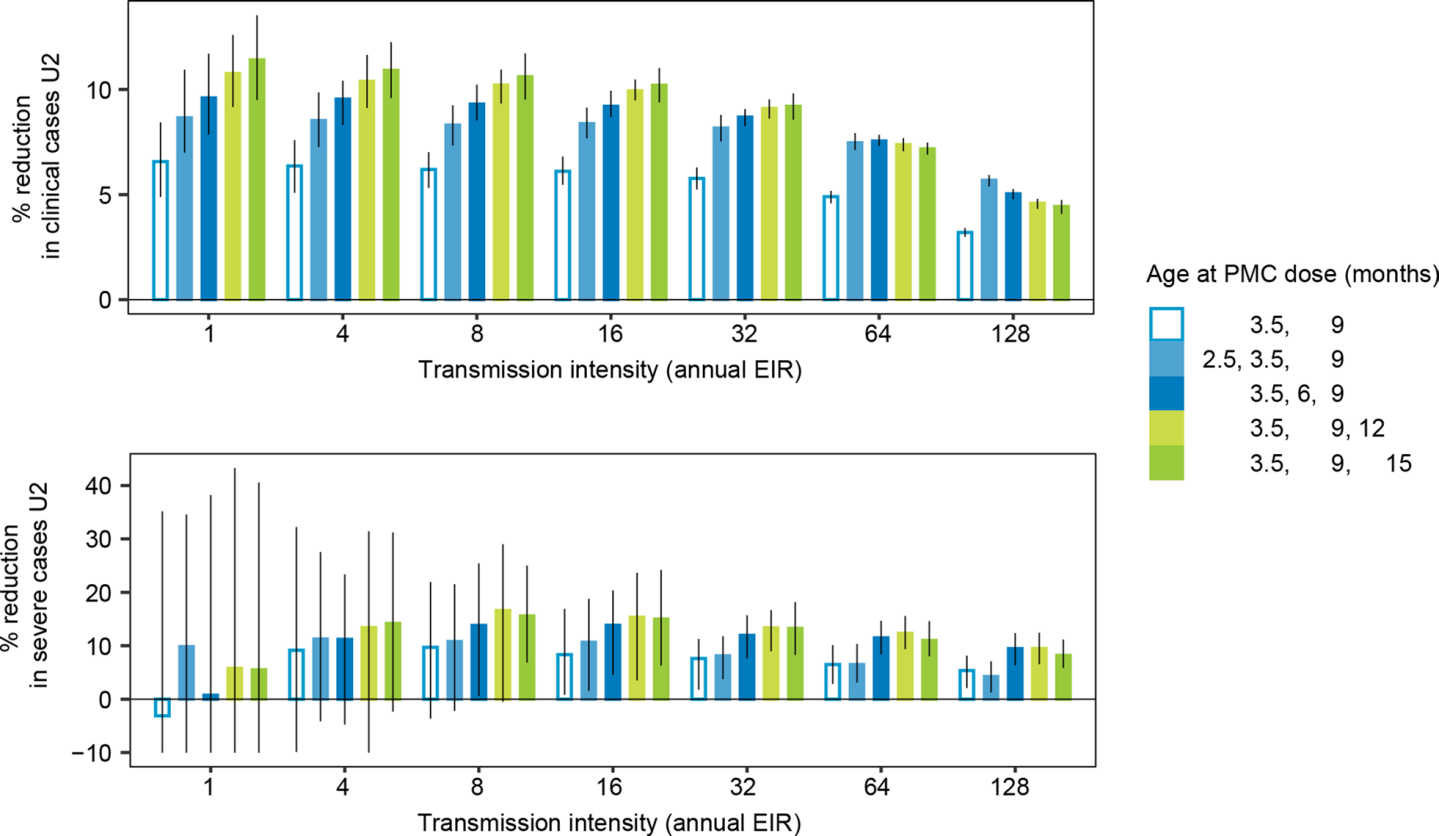
Relative importance of first PMC dose in PMC-3 when omitted or shifted by transmission intensity and disease severity. Projected relative reduction in clinical cases (top) and severe cases (bottom) in children under the age of 2 years. PMC-3 was simulated with 80% coverage for each dose. Prediction intervals were truncated at − 10%

Overall, three doses were more impactful than two doses, indicating that the dose at 2.5 months does provide additional protection against clinical malaria although to a lesser extent against severe malaria (Fig. 3). This finding is likely influenced by assumptions on maternal antibody protection in the model, which has a stronger effect on severe than on clinical malaria (Additional file 1: Fig A1.3.5). Shifting the 2.5-month dose to older ages resulted in an increased reduction in cases; the amount of increase varied by disease severity and transmission intensity. For clinical malaria, timing this dose to later ages (6, 12 or 15 months) was more impactful at low-to-high transmission, but not at very high transmission (EIR ≥ 62 ibpa). For severe malaria, shifting the dose to 6 or 12 months of age increased impact across all transmission levels, whereas a shift to 15 months reduced its impact, especially at very high transmission (Fig. 3).

### Impact of additional PMC doses with or without RTS,S in children under 2 years of age

**Fig. 4:**
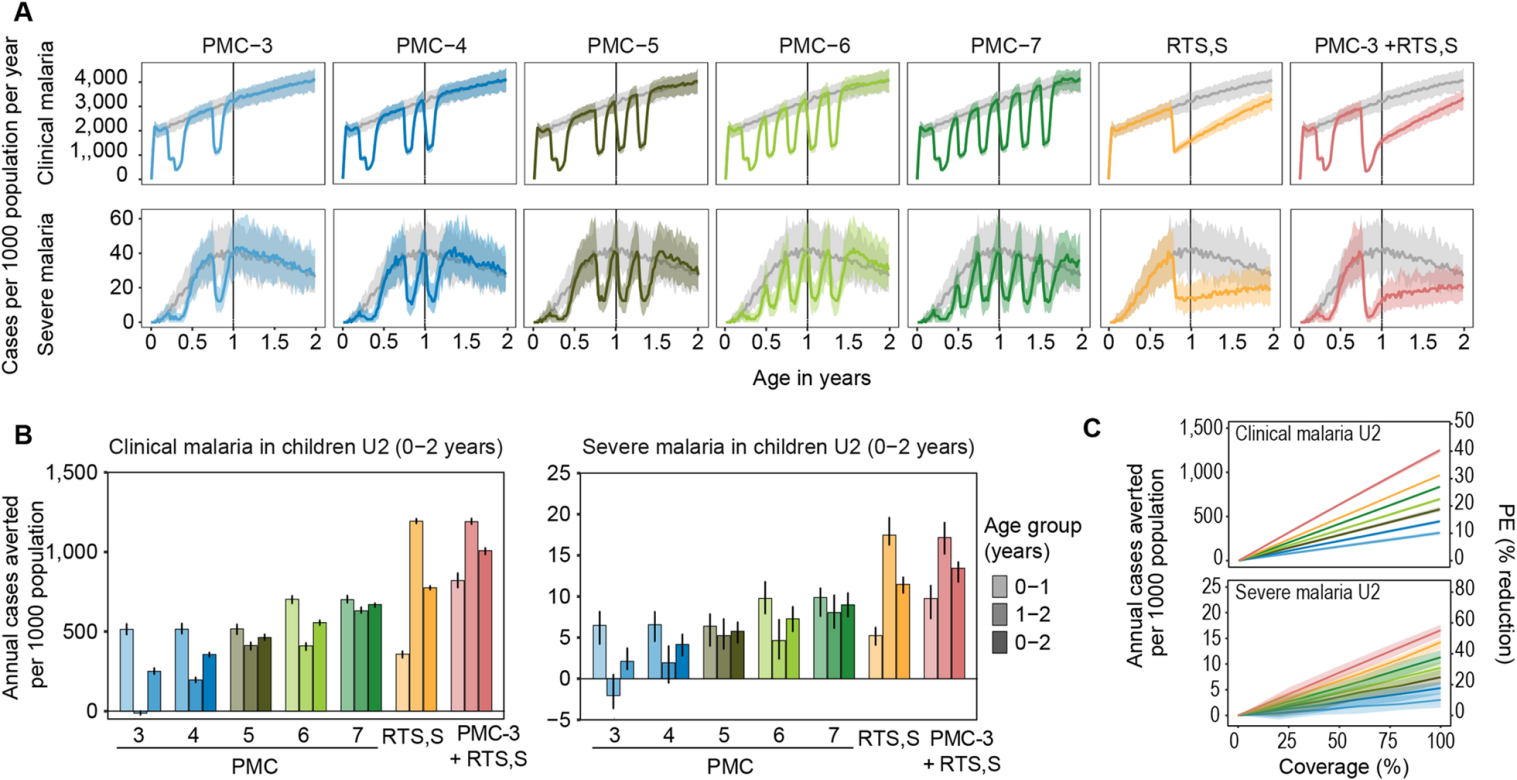
Predicted impact on malaria case incidence of PMC schedule with and without RTS,S in children under the age of two years at high transmission intensity. **A** Clinical and severe case incidence per 1000 population per year by age in the absence of PMC or RTS,S (gray line) or with PMC at various schedules or RTS,S, at 80% coverage. The solid line shows median and shaded area shows 90% PI. **B** Number of cases averted per 1000 population per year by PMC-RTS,S scenario at 80% coverage for children 0–1 and 1–2 years and U2 (0–2 years). The bar shows median and the error bars show 90% PI (relative reductions: Additional file 1: Fig A1.2.8). **C** Impact on clinical and severe cases of PMC-RTS,S scenarios at varying coverage levels, with median number of cases averted per 1000 population per year on the primary, y-axis and PE on the secondary y-axis. Projections varying by levels of transmission shown in Additional file 1: Fig A1.2.9

Five PMC schedules (Fig. 1B) were compared to each other as well as to RTS,S in a simulation with high transmission (EIR = 32 ibpa, 3000 cases per 1000 population U2), assuming a constant target coverage of 80% for each dose of PMC and for RTS,S (Fig. 4A). Aggregated clinical and severe cases averted for children 0–2 years (children U2) and disaggregated into 0–1 and 1–2 years are shown in Fig. 4B.

In children U2, RTS,S was projected to avert the most cases, followed by PMC scenarios based on how many doses were given. PMC with three to seven doses in the first 18 months of age (PMC-3, PMC-4, PMC-5, PMC-6, and PMC-7) averted on average 251 to 669 clinical cases per 1000 children U2 (PE ranging from 8.1 to 21.6%), compared to 774 (25.1%) cases averted by RTS,S and 1003 (32.6%) by the combination of RTS,S and PMC-3. For severe malaria, between two and nine cases were averted per 1000 children U2 (PE 7.3−31%) across the same PMC scenarios, compared to eleven (PE 39.8%) cases averted by RTS,S, and thirteen by the combination (PE 46.4%). Overall, PMC averted most cases during the first year of life, and RTS,S after the first year (Fig. 4B). For all the PMC schedules tested, RTS,S combined with PMC had a complementary effect, even under PMC-7, where overlap was greatest (Additional file 1: Fig A1.2.5).

The maximum PE reached in children U2, assuming a 100% coverage, ranged from 10 to 41% across the PMC-RTSS scenarios for clinical malaria and from 10 to 57% for severe malaria (Fig. 4C).

### Operational impact of PMC and RTS,S in Southern Nigeria

**Fig. 5:**
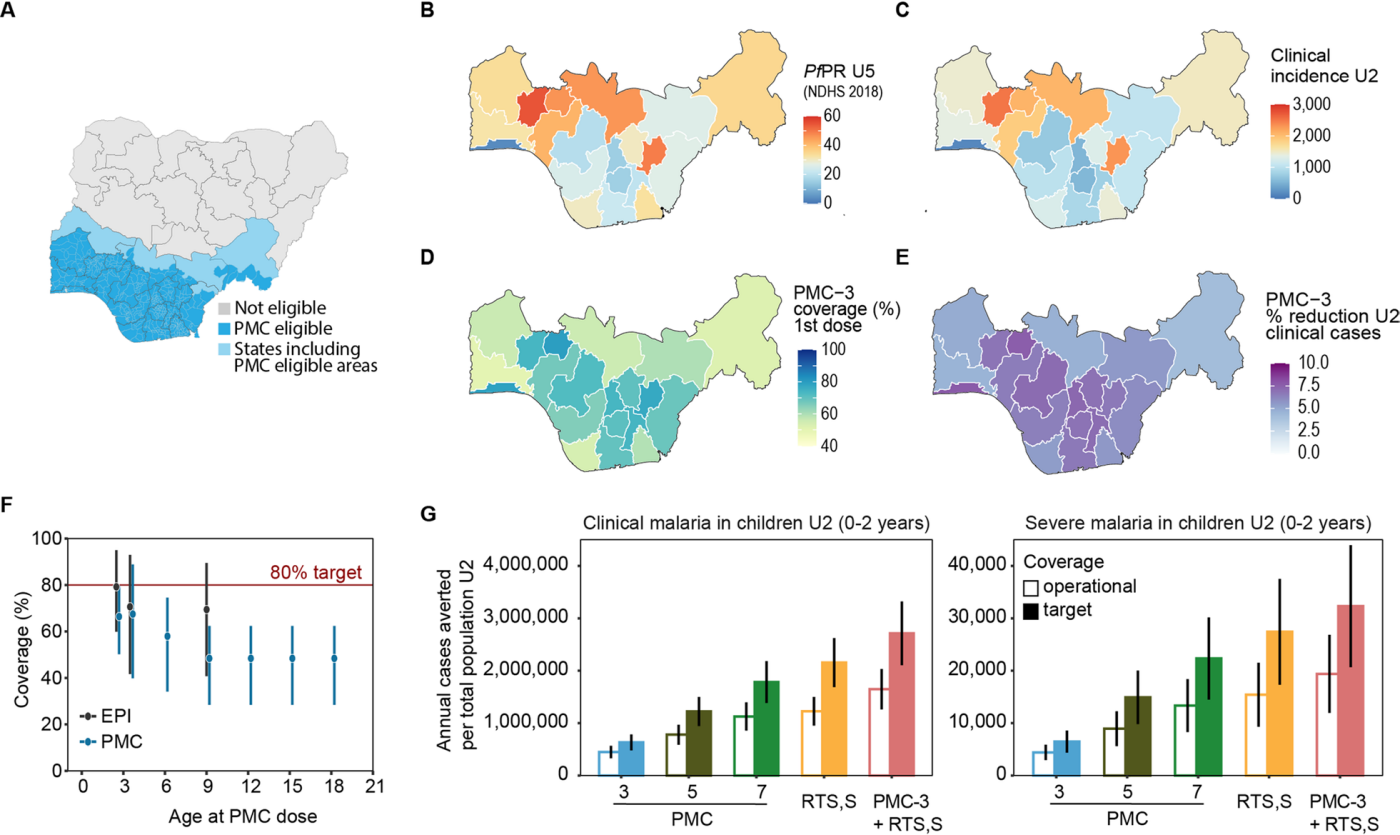
Projected intervention impact of PMC and/or RTS,S at operational and target coverage for Southern Nigeria. **A** Nigerian States with PMC-eligible areas (n = 20 States). **B–E** Maps of Southern Nigeria showing (**B**) *Pf*PR in children U5 according to rapid diagnostic test from the NDHS 2018; (**C**) simulated clinical cases per 1000 population per year in children U2; (**D**) coverage of first PMC dose by State; (**E**) predicted relative reduction in clinical cases in children U2 by State. **F** Coverage by potential PMC touchpoint, showing mean and range across 20 States in Southern Nigeria. See "Methods" section on estimation process for likely PMC coverage. **G** Annual clinical cases averted in children U2 at operational coverage and at target coverage (80%) for five PMC and/or RTS,S scenarios

A second set of simulations was run where transmission intensity, seasonality, and PMC coverage corresponded to the 20 States in Southern Nigeria that include PMC-eligible areas (Fig. 5A). Malaria prevalence from the NDHS 2018 and *Pf*PR_U5_-EIR relationship from the previous simulations were used to obtain annual EIR values for each State. Malaria prevalence ranged between 3.4 and 54.9% across States (mean 30.3%) and matched EIRs ranged from 1.1 to 27.5 ibpa (mean 11.9 ibpa) (Fig. 5B, Additional file 1: Fig A1.4.3). The simulated malaria incidence in the absence of PMC or RTS,S ranged between 152 and 2,510 clinical cases and between 0.735 and 33.71 severe cases per 1000 children U2 per year across the States (Fig. 5C). For a population of 6.5 million children U2, estimated for 2019 in Southern Nigeria, the projected malaria burden was around 7.6 million clinical and 69,000 severe cases in one year. The presented malaria case estimates include untreated and unreported cases. The operational PMC coverage was based on State-level estimates of EPI coverage reported in NDHS 2018 (mean 73.1%, range 40.8–95% for 3 doses across States), and downscaled to account for expected gaps between immunization and PMC coverage [3] (mean 60.8%, range 28.5–88.8%) (Fig. 5D,F).

At operational coverage, PMC-3 was projected to avert annually on average 447,258 (95%CI 329,041–565,476) clinical and 4,409 (95%CI 2,950–5,868) severe cases in the U2 population (Fig. 5E,G). As expected, the relative impact was strongly correlated with coverage, and the total number of cases averted was highest in States with higher population and malaria burden. PMC-5 was projected to avert nearly twice as many cases as PMC-3, with 779,263 (95%CI 589,395–969,131) clinical and 8,931 (95%CI 5,619 − 12,244) severe cases averted. PMC-7 was projected to avert 1,125,500 (95%CI 856,435–1,394,566) clinical and 13,360 (95%CI 8,314 − 18,404) severe cases. In comparison, RTS,S was projected to avert 1,225,010 clinical cases (95%CI 951,960–1,498,060) and 15,419 (95%CI 9,310−21,527) severe cases. Finally, the combination of RTS,S plus PMC-3 was projected to avert 1,647,743 clinical cases (95%CI 1,262,623–2,032,864) and 19,394 (95%CI 11,946−26,841) severe cases annually in children U2, a protective efficacy against clinical cases of 23.4% (95%CI 21.1–25.7%) and against severe cases of 29.9% (95%CI 27.3–31.6%).

If coverage were to increase to target levels of 80%, more cases could be averted (Fig. 5G). For instance, an increase in the mean coverage of PMC-3 from 61 to 80% averted 41–46% more cases with additional 185,014 clinical and 2063 severe cases averted per year per population U2. The additional cases averted when increasing coverage from operational to target levels increased with the number of doses. For PMC-5, an increase in coverage from 56 to 80% averted on average 442,936 additional clinical cases per year and 5993 additional severe cases. For PMC-7 an additional 658,421 clinical and 8983 severe cases were averted per population U2 per year when coverage increased from 55 to 80%. A similar trend was projected for RTS,S with 930,503 additional clinical and 12,004 severe cases averted, at target compared to operational coverage and for the combination of RTS,S and PMC-3 with additional 1,066,371 clinical and 12,926 severe cases averted, corresponding to protective efficacies of 36.6% and 46.4% against clinical and severe cases, respectively (Fig. 5G).

## Discussion

Using an individual-based mathematical model, allowed to address uncertainties about scheduling PMC alone or in combination with malaria vaccines like RTS,S [12] to provide additional evidence and improved understanding required to accelerate policy adoption of PMC in countries with high malaria burden in children. Simulated PMC with up to seven doses during the first eighteen months of age showed an added benefit in impact against clinical and severe malaria. In children under two years of age, at least seven doses of PMC at high coverage would be required to avert as many cases as malaria vaccination with RTS,S. Across all the PMC schedules tested, there was a complementary effect between PMC and RTS,S, providing a continuum of protection, even with PMC-7 where overlap was high. While rollout at scale could potentially avert thousands of cases per year, in settings like Southern Nigeria substantial burden is likely to remain even if both PMC-3 and RTS,S are distributed and target coverage is reached.

Across varying transmission levels, PMC and RTS,S averted the most clinical cases under moderate to high transmission conditions and fewer at low or very high transmission levels. This trend in impact by transmission is consistent with a previous modelling study on PMC [41], on RTS,S [27], and with secondary analysis of RTS,S phase 3 trial data [42], due to increased rebound effects at very high transmission. The effectiveness and timing of the first three doses of PMC was found to be especially sensitive to the intensity of transmission since the malaria burden peaks at different ages for clinical and severe malaria due to dynamics between exposure and immunity acquisition [43]. Hence, to identify the appropriate PMC schedule with the greatest potential epidemiological impact, a good understanding of local clinical and severe malaria incidence by age is needed.

High coverage is also necessary to maximize epidemiological impact. Reaching high coverage for every dose is unlikely, especially as children get older. In many sub-Saharan African countries, vaccination coverage through the EPI system remains below the target of 80% [44, 45] and tends to decline with the child’s age [46] or with supplemental touchpoints such as Vitamin A supplementation [47]. The modelling results suggest that limited coverage with PMC could be compensated for by increasing the number of doses, whereas in practice whether to increase coverage or number of doses likely differs in cost-effectiveness and operational feasibility. Local assessment in health facilities will be crucial to obtain information on feasible coverage of PMC in the first two years of life that, when combined with age-incidence data, can inform decisions on appropriate PMC schedules.

Decisions on the appropriate PMC schedule and whether to combine PMC and malaria vaccination will also depend on community acceptance and tolerance, which were not included in the model. Interestingly, a community-accepted PMC implementation itself may improve immunization coverage [48]. It is possible, however, that in some communities, too many doses might be perceived as an ‘overload’, since ‘children already receive so many vaccines’ [49, 50].

The presented analysis includes simplifying assumptions that are relevant to the interpretation of the results. First, the modelled efficacy of PMC-3 in infants was matched to pooled estimates from various clinical trials across Africa conducted between 2000 and 2013 [7]. This allowed us to make generalizable projections sufficient to describe trends but might underestimate impact as some clinical trials showed higher efficacy [51]. Conversely, the simulated effect size in severe cases was larger than the combined effect observed in two trials [52, 53]; however, obtaining sufficient statistically-powered effect estimates on severe cases in clinical trials is challenging and confidence intervals are often too wide to give a clear indication on impact in severe cases [54].

Second, it was assumed that intervention efficacy did not vary by age or number of doses, as clinical studies showed a relatively consistent pattern of four to five weeks of protection per dose of SP [38]. If chemoprevention efficacy changes with age [43], the results would be overestimated, particularly for scenarios with PMC into the second year of life.

Third, the PMC coverage after nine months of age was assumed to stay constant, which likely results in too optimistic operational effectiveness projections of PMC if coverage at older ages further declines. For example, a study in Ghana observed low uptake of the malaria vaccine booster at 24 months of age despite overall high uptake [55]. More data is needed to inform realistic coverage estimates in the second year of life.

Fourth, parasite resistance against SP is widespread in East Africa, possibly growing in West Africa, [56, 57], and poses a great concern for country programs in their decision to adopt PMC [58, 59]. By not including resistance explicitly and using efficacy data from the first decade in 2000 to calibrate PMC’s effect size, the predicted impact of PMC might be overestimated. However, the relationship between chemoprevention use and resistance is complex [60], and continued monitoring will be crucial during and after the implementation of PMC, as is being done for SMC [61].

Lastly, biological, and immunological dynamics that might affect intervention efficacy such as the acquisition of partial immunity, nutritional status, or maternal antibody protection remain highly uncertain. For instance, one modelling study found maternal antibody protection to have a large effect on PMC impact estimates [41], whereas in the presented model, maternal antibody assumptions mostly influenced the impact on severe malaria (see Additional file 1: A1.3).

Despite these limitations, these modelling results provide useful additional evidence on the potential and relative benefit of PMC with or without RTS,S that is generalizable across settings and can be helpful in informing pilot studies, strategic considerations of PMC adoption in countries, as well as subsequent modelling studies. In practice, decisions on where and how to implement PMC with or without RTS,S will be highly dependent on costs, available funding, existing malaria policies, health system preparedness as well as community acceptance. Countries that decide to implement PMC and or the malaria vaccine should collect disaggregated age-specific data in children under the age of five years to monitor the actual impact of the interventions in reality. These data will also allow modellers to review and update their model assumptions with real-life effectiveness data for improved impact predictions. Finally, reducing malaria burden during early childhood will take a holistic approach, and strengthening health systems in high-burden countries remains a fundamental prerequisite for reaching elimination [62].

## Conclusion

PMC can reduce substantial clinical and severe cases of children U2 in areas with high burden and perennial transmission. Nevertheless, its impact will be limited by the operational coverage, and the number of feasible touchpoints. Identifying the age groups that are most vulnerable to malaria disease, and are most likely to uptake at high coverage, in a given setting is crucial for determining which PMC schedule or potential combination with RTS,S, would be most appropriate.

## Supplementary information

**Additional file 1:** Methodological supplement and additional results. 

## Data Availability

The dataset analysed and generated via simulations as well as analysis scripts are available from GitHub: https://github.com/numalariamodeling/PMC_modeling_publication_2023.

## Abbreviations

DTP: Diphtheria, tetanus, pertussis
EIR: Entomological inoculation rate
EPI: Expanded Program on Immunization
GFATM: Global Fund to Fight Aids, Tuberculosis and Malaria
ibpa: Infectious bites per person per annum
IPTi: Intermittent Preventive Treatment in infants
NDHS: Nigeria Demographic Health Survey
PE: Protective efficacy defined as relative reduction
*Pf*PR: *Plasmodium falciparum *prevalence rate
PMC: Perennial malaria chemoprevention
U5: Under 5 years of age (U1, U2 under 1 or 2 years)
SMC: Seasonal malaria chemoprevention
WHO: World Health Organization
