# Supplementary material for: Perennial malaria chemoprevention with and without malaria vaccination to reduce malaria burden in young children: a modelling analysis
Source: Malar J. 2023 Apr 24;22:133. doi: 10.1186/s12936-023-04564-9 (PMC10124689; doi:10.1186/s12936-023-04564-9)
Supplement: Supplementary file 1 — Additional file 1: Methodological supplement and additional results. [file 12936_2023_4564_MOESM1_ESM.docx]

Additional file 1

Perennial malaria chemoprevention with and without malaria vaccination to reduce malaria burden in young children: a modeling analysis

**Table of contents**

[**A1.1: PMC and malaria vaccine efficacy calibration and validation** 1](#_Toc130051863)

[*PMC* 1](#_Toc130051864)

[*Malaria vaccine* 4](#_Toc130051865)

[**A1.2: Geographic-agnostic model** 5](#_Toc130051866)

[*Transmission seasonality* 5](#_Toc130051867)

[*Transmission intensity, prevalence, and incidence relationships* 5](#_Toc130051868)

[*Additional result figures* 6](#_Toc130051869)

[**A1.3: Sensitivity analysis, exploration of parameter uncertainty** 11](#_Toc130051870)

[Completeness of PMC doses 11](#_Toc130051871)

[Clinical treatment coverage 13](#_Toc130051872)

[Age-varying clinical treatment coverage 13](#_Toc130051873)

[Maternal antibody protection 14](#_Toc130051874)

[**A1.4: Country application to Southern Nigeria** 16](#_Toc130051875)

[**References** 18](#_Toc130051876)

## **A1.1: PMC and malaria vaccine efficacy calibration and validation**

### *PMC*

The simulation was based on a geographic-agnostic model with 32 infectious bites per person per annum (ibppa) and clinical case management at 60% with a single dose of PMC administered at 14 weeks of age at 100% coverage (for efficacy). We used the EMOD vaccine campaign event to model PMC, which allowed us to specify custom waning efficacy curves^^[[1]](#footnote-1)^^. An empirically derived reference efficacy curve of sulfadoxine-pyrimethamine (SP) based on a Ghana IPTi trial was used to estimate initial efficacy and decay patterns [[1]](https://www.zotero.org/google-docs/?ibrF6w). In the simulated population, 100 drug response groups were defined to model variation in the initial efficacy of the drug, defined as the maximum proportion of blood-stage parasites killed by the drug. The parameter values were drawn from a normal distribution with a mean of 0.8, a standard deviation of 0.025, and truncated at 0.75 and 0.9, arbitrarily chosen. The efficacy was held constant for a specific time before decaying exponentially and parameters (*Box_Duration*, *Decay_Time_Constant)* were fitted using values between 28 and 34 days and between 14.4 and 21.6 respectively. The protective efficacy against clinical malaria per week following simulated administration of the single dose of PMC was calculated and matched to the reference SP efficacy curve [[1]](https://www.zotero.org/google-docs/?IhyXpj) using the least sum of squares. The estimated parameters were used in a follow up simulation with varying transmission intensities (annual EIR <10 to 256 infectious bites per person per annum (ibpa)) to compare resulting protective efficacies in children 0-12 months to reported protective efficacy estimates in infants from previous IPTi trials [[2,3]](https://www.zotero.org/google-docs/?EvFatD) (Fig A1.1.1).


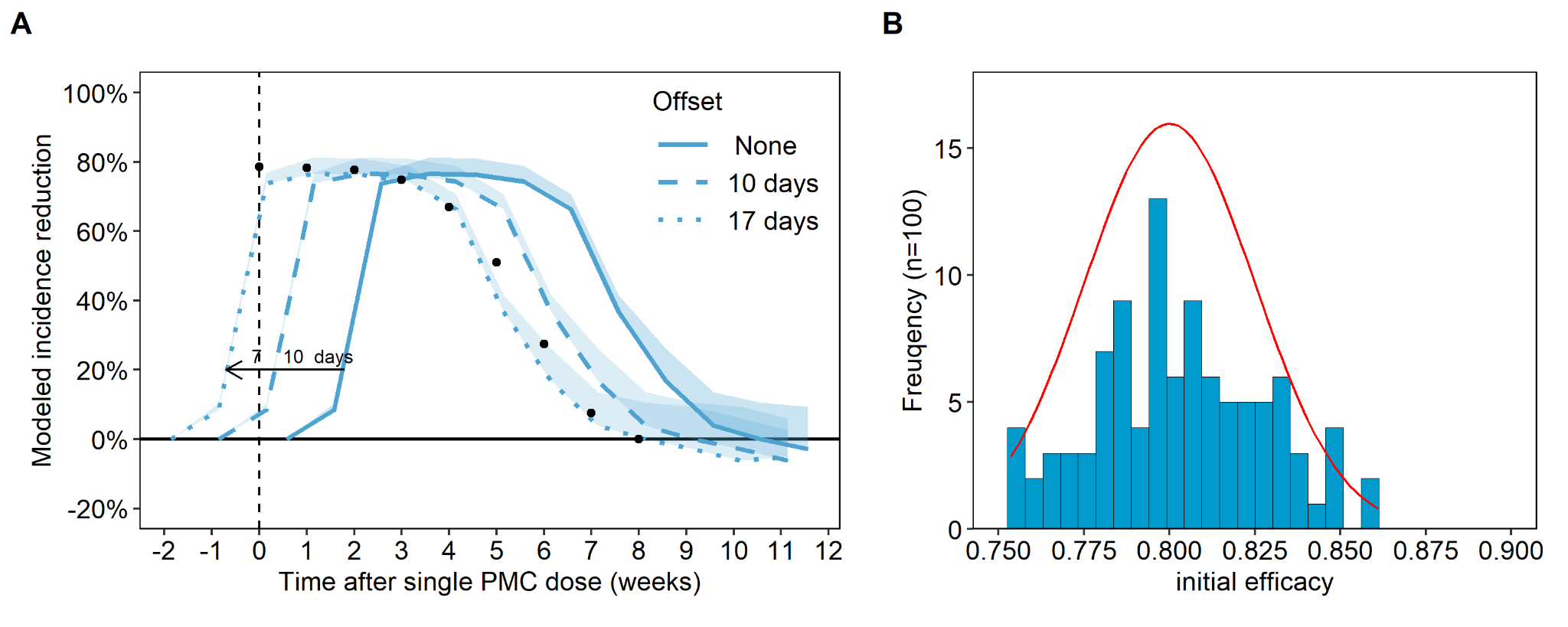


**Fig A1.1.0: A)** Time offset in efficacy curve of a single dose of SP for clinical malaria. For each age touchpoint, an offset of 10 days was included in the simulation. To match the reference curve with immediate efficacy at around 80%, an additional offset of 7 days was required. **B)** Distribution of initial efficacy of single PMC dose for 100 dose-response groups in the simulated population (mean = 0.8, sd = 0.025, min =0.75, max = 0.9).


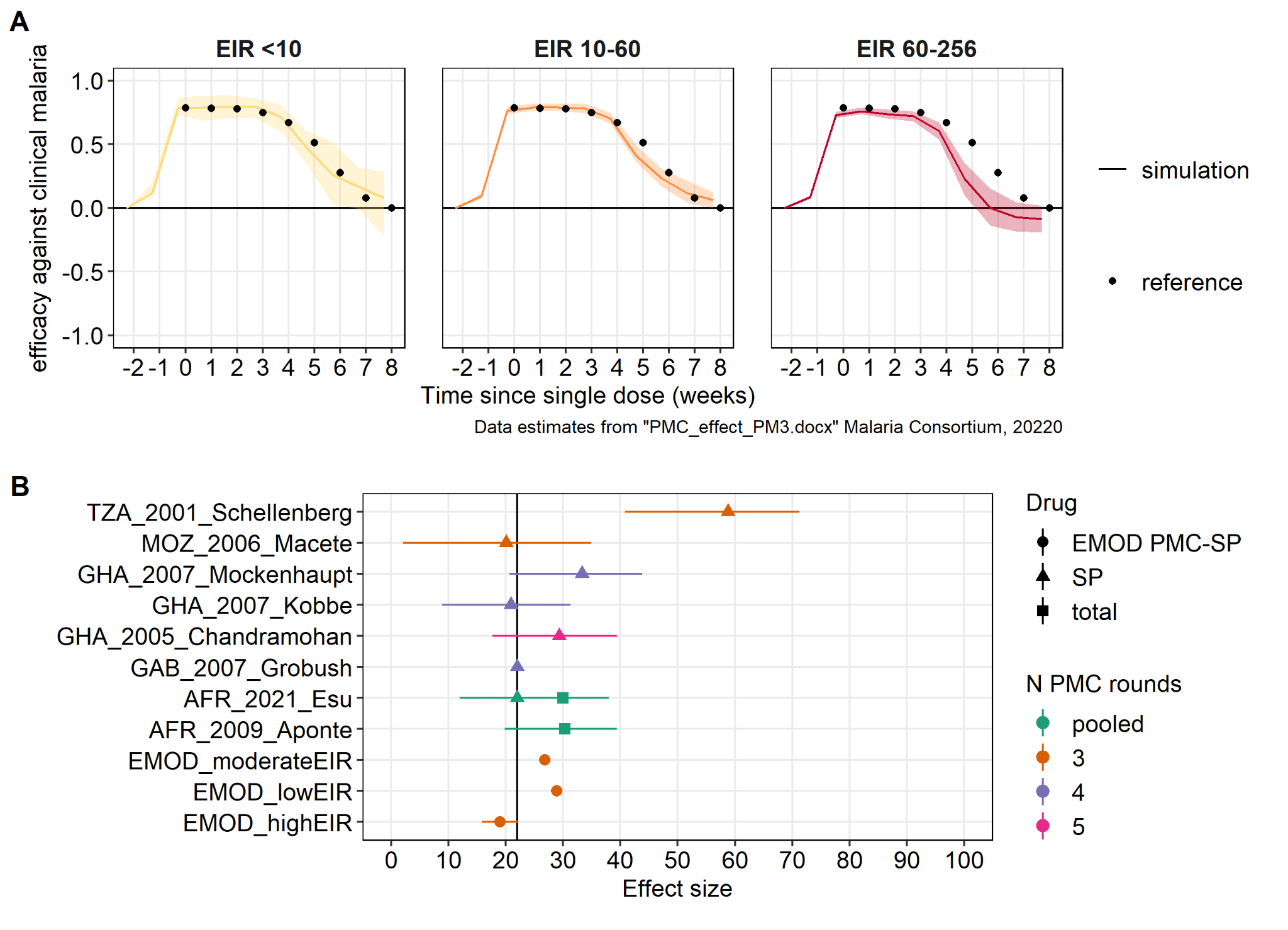


**Fig A1.1.1: A)** Efficacy curve of a single dose of SP for clinical malaria by varying levels of transmission intensity. Reference points smoothed estimates based on averaged effect across four doses in children less than 15 months in Ghana IPTi 2005 trial [[1,4]](https://www.zotero.org/google-docs/?QNKdcP). Simulations were adjusted with an offset of 7 days since no immediate parasite clearance was simulated. **B)** Comparison of protective efficacies for clinical malaria across clinical trials, pooled analysis, and EMOD simulations. Effect sizes per trial were obtained from [[2]](https://www.zotero.org/google-docs/?ZEwG9i) as well as source publications.

*Validation of modeled PMC-SP efficacy in Ghana IPTi trial*

Between 2000 and 2004 a PMC clinical trial was conducted in Navrongo [8], a demographic sentinel site in Northern Ghana. At the time of the study, the first line treatment was chloroquine, and second line treatment SP. Transmission in the study area in 2002 was very high (418 ibppa) and highly seasonal with a transmission peak in October. Children born between September 2000 and July 2002 were enrolled in the study and given SP or placebo at the age of two, three, nine, and twelve months and followed up until July 2004. The primary endpoint was malaria incidence in children attending health facilities or hospitals. The study found a reduction of 24.8% (14.3 - 34.0%) in clinical cases in infants as for PMC-4 [[4]](https://www.zotero.org/google-docs/?AWqajC).

An EMOD simulation was set up to mimic the IPTI trial in Ghana, with individuals assigned to either a placebo or a treatment group at three months of age and followed up for four years. No conclusive data were found for the drug efficacy of chloroquine in the early 2000s in Northern Ghana, and an effective treatment coverage of 40% for children under the age of five years was assumed. The effective treatment coverage was selected under consideration of high resistance against chloroquine in 2003 [[5,6]](https://www.zotero.org/google-docs/?vKffvk), as well as case management numbers in the study area in the early 2000s [[7]](https://www.zotero.org/google-docs/?i7mBMF) (i.e. around 36% care seeking at government clinics, 93% of action taken within 24 hours, and 56% appropriateness of prescription). Reported EIR values from an entomological study [[8]](https://www.zotero.org/google-docs/?n4OM7g) in the same area and time as the IPTi trial informed the transmission seasonality in the simulation. However, to be able to match the seasonal trend in cases reported in the IPTi trial, the monthly EIR values had to be shifted back by one month. Additionally, a scaling factor on the transmission intensity was fitted to the reported monthly case data for the placebo group using least sum of squares, resulting in an EIR of 290 ibpa (Fig A1.1.2A). The reported prevalence at two timepoints during the IPTi trial was used for cross-validation (Fig A1.1.2B)


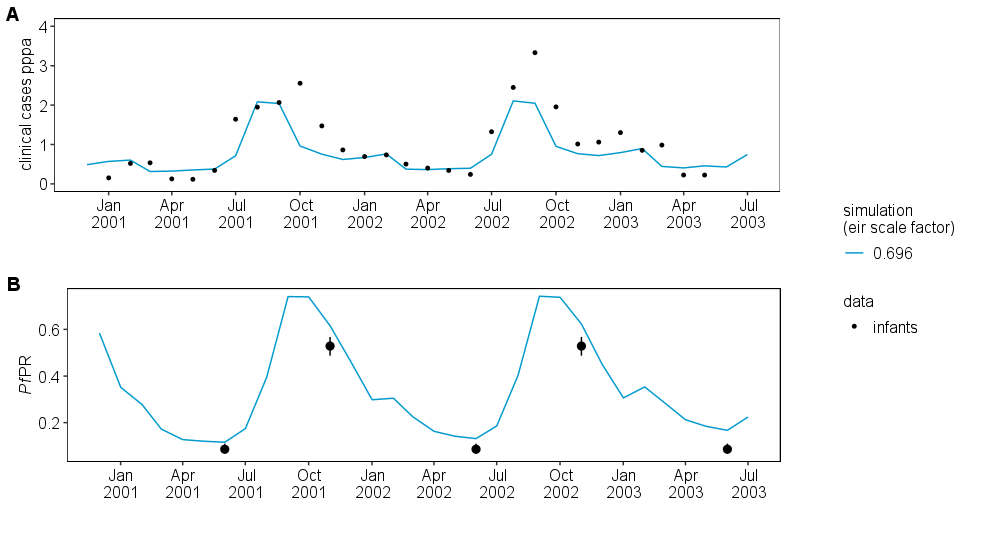
**Fig A1.1.2:** Model calibration to Navrongo, Northern Ghana**. A)** Simulated and observed clinical malaria in the placebo group. **B)** Simulated and observed prevalence in the placebo group, not fitted. Data obtained from [[4]](https://www.zotero.org/google-docs/?Wk00al).

In the simulation, PMC was administered to the treatment group as described in the study and the simulated total clinical cases compared to reported clinical cases for the period after each dose as well as for all doses combined (Fig A1.1.3).


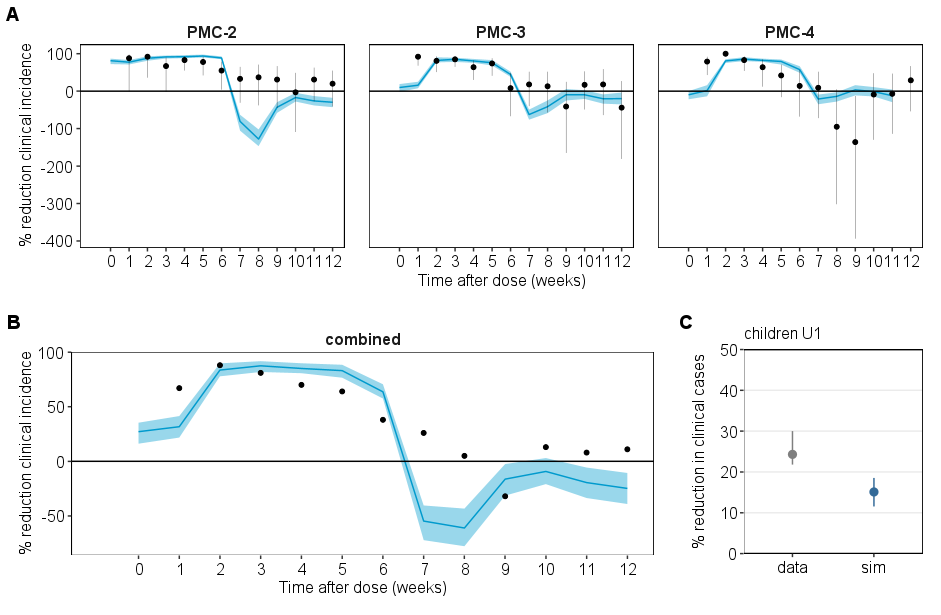


**Fig A1.1.3:** Simulated and observed PMC efficacy per dose in Navrongo trial. **A)** per PMC dose. **B)** averaged across doses. **C)** PE for clinical cases aggregated for children U1. Data source for clinical trial in study cohort obtained from [[4]](https://www.zotero.org/google-docs/?NKtpaB). Simulation results shown with mean and 90% prediction interval based on stochastic runs. In this figure, the number in PMC-2, PMC-3, and PMC-4 denotes the timing of PMC dose (age 3,9,12 months of age).

### *Malaria vaccine*

In EMOD pre-erythrocytic vaccines are modeled with a proportionate reduction in the force of infection following an exponential decay. The malaria vaccine RTS,S was modeled, which has been previously calibrated to phase-3 trial data [[9]](https://www.zotero.org/google-docs/?v4o6pj), and published with a detailed description of methods [[10]](https://www.zotero.org/google-docs/?FN2N47). The best-fitting parameters were an initial efficacy of 80% immediately after the third dose, assuming no partial effect of earlier doses, and an exponential decay over 13.5 months [[10]](https://www.zotero.org/google-docs/?kVSxHm).

*
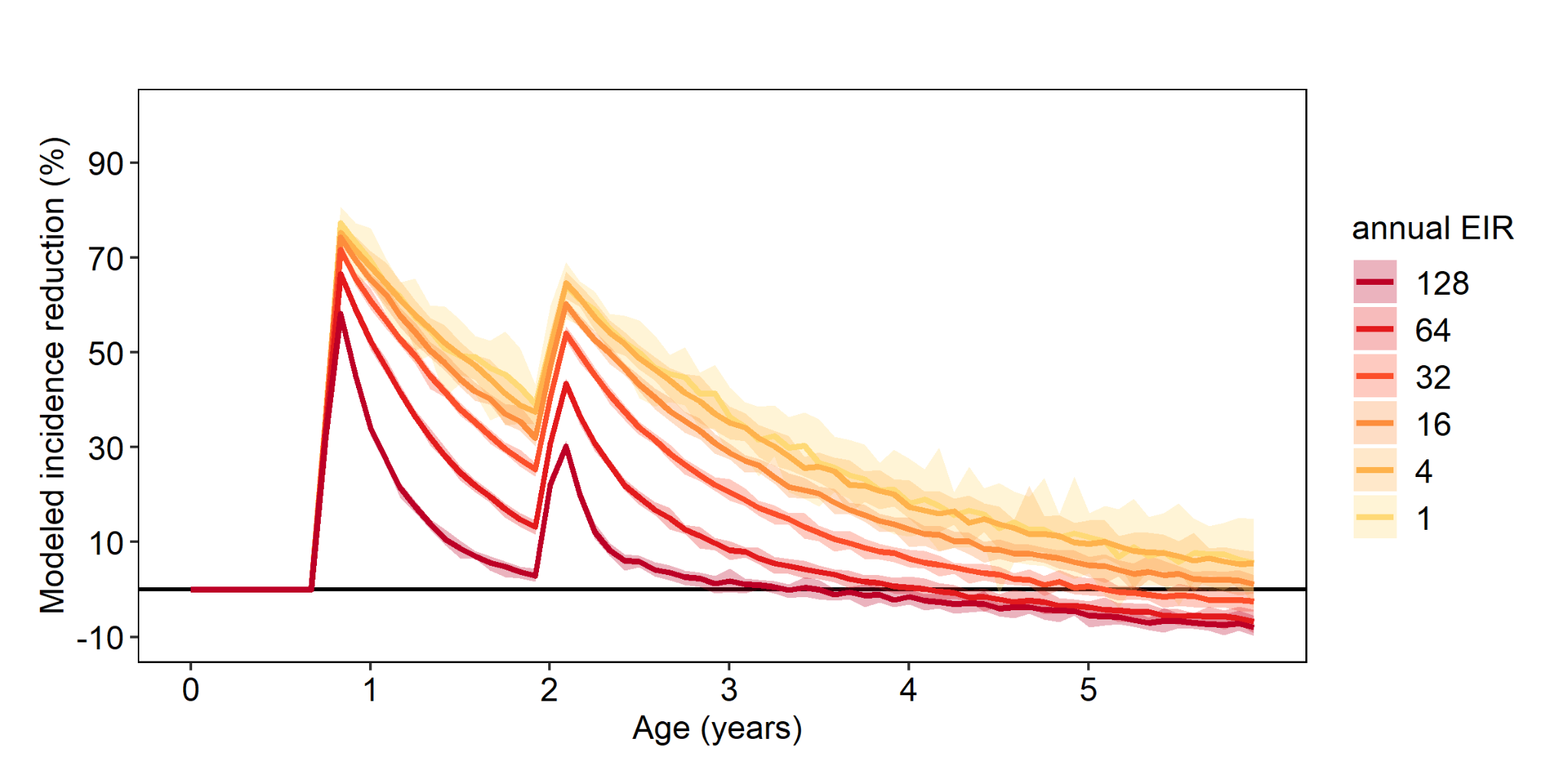
*

**Fig A1.1.4:** Intervention efficacy of the modeled malaria vaccine RTS,S on clinical malaria over age and by transmission intensity.

## **A1.2: Geographic-agnostic model**

### *Transmission seasonality*

Monthly transmission values determine malaria exposure of each of the 12 birth cohorts simulated and were presented in the results as uncertainty intervals. To obtain realistic seasonality patterns for perennial transmission setting, monthly EIR values were extracted from the Nigeria model calibration described in Ozodiegwu et al [[11]](https://www.zotero.org/google-docs/?qNXAxe). The monthly EIR values were categorized based on the start of the season, the variability across months and the number of consecutive high/ low transmission months in relation to the annual average. Depending on the number of peaks and variability, three seasonality patterns were defined and rescaled to different annual EIR levels (Fig A1.2.1). To constrain computational costs and limit the range of prediction intervals, only ‘seasonality pattern 1’ was used in the main manuscript.


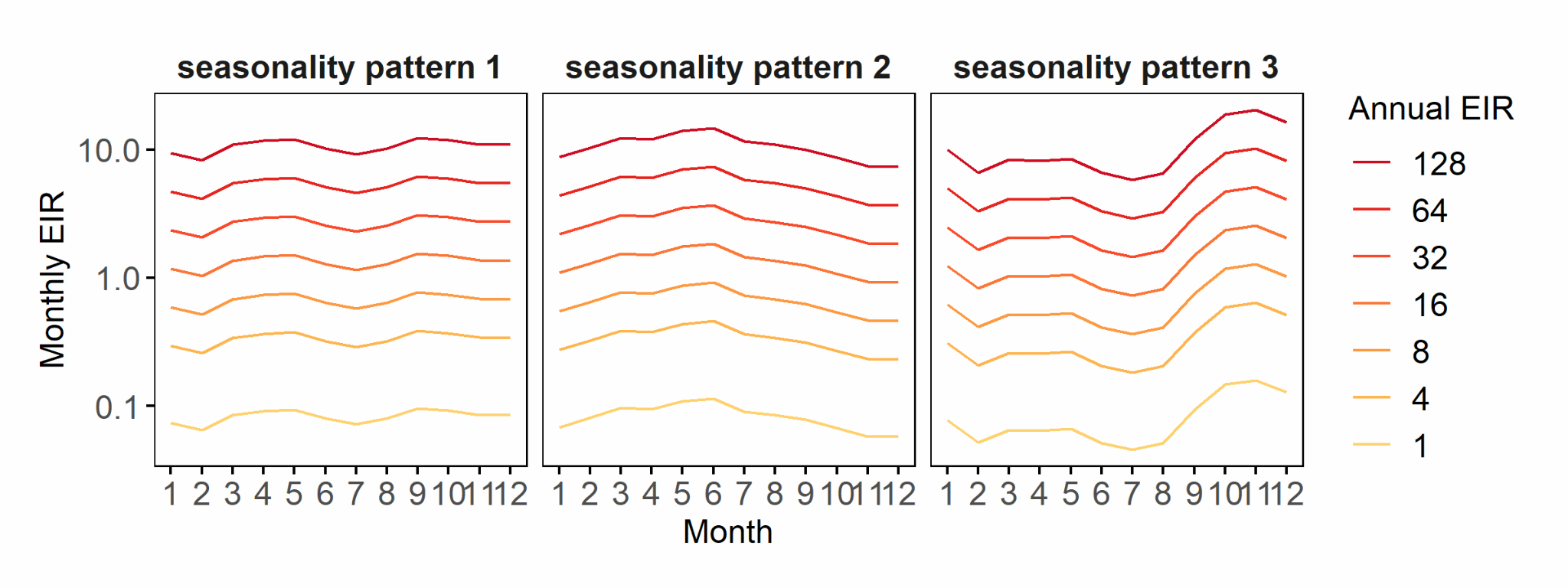


**Fig A1.2.1:** Seasonality patterns in perennial transmission settings corresponding to Southern Nigeria. Seasonality patterns derived from fitted monthly EIR estimates from Nigeria High Burden to High impact model, calibrated to monthly malaria incidence [[11]](https://www.zotero.org/google-docs/?lYqxjR). Monthly EIR on y-axis shown on log-scale. Only ‘seasonality pattern 1’ was used in the main manuscript.

### *Transmission intensity, prevalence, and incidence relationships*

The geographic-agnostic model ran for EIRs between 1 and 128 ibpa and fixed treatment coverage levels without additional interventions to assess trends in malaria outcomes (*Plasmodium falciparum* prevalence rate (*Pf*PR), clinical and severe malaria incidence). Overall, as transmission increased so did the malaria outcome metrics, however, to varying extents depending on age group. Malaria prevalence reached higher levels in older than in younger children, with a maximum of 60%-80% at 128 ibpa. The maximum clinical incidence ranged between 4-5.5 episodes per person per year and was highest for children U5, U2, and U1, whereas cases in children U10 clinical incidence plateaued at around 32 ibpa. The maximum severe incidence ranged between 0.02 to 0.11 episodes per person per year and for EIR levels higher than 16 ibpa was highest in children U1 and U2 and substantially lower in children U5 and U10 (Fig A1.2.3).


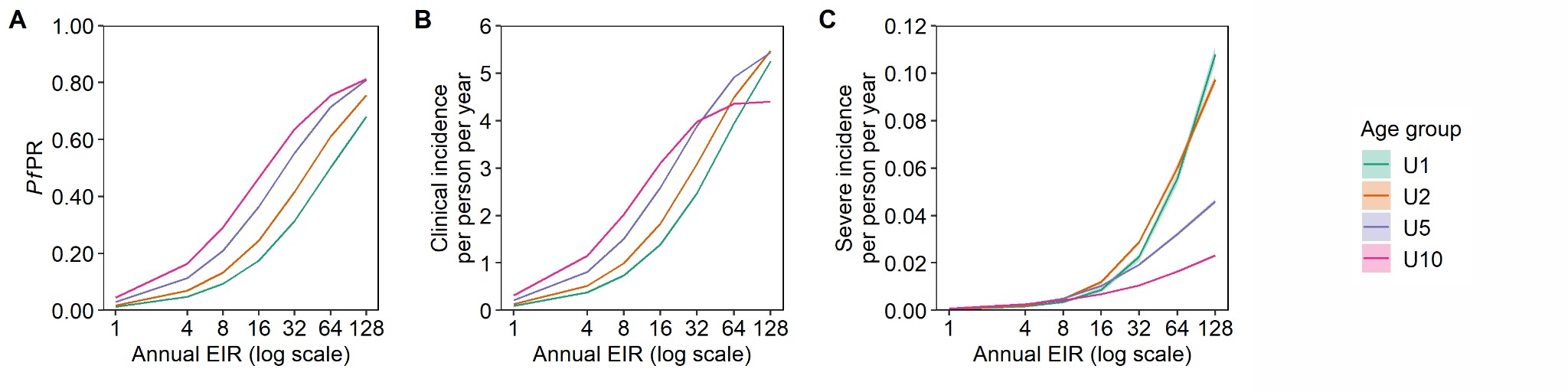


**Fig A1.2.2: Prevalence, incidence curves by transmission intensity and age group.**

**A)** *Pf*PR - EIR curve by age group. **B)** Clinical incidence - EIR curve by age group. **C)** Severe incidence - EIR curve by age group. In A-C, the annual EIR refers to the EIR in the total population. Predictions based on the geographic-agnostic simulation model without any other malaria control intervention except for effective treatment coverage at 60% for clinical and 80% for severe cases.

### *Additional result figures*

The geographic-agnostic model ran for ten years with case management only, or additional PMC, or additional PMC + RTS,S. At very high transmission intensity, the number of clinical cases start to drop at around five years of age, whereas at lower levels of transmission, clinical cases gradually increase and decrease with a high number of cases until at least ten years of age. Transmission intensity affects the number of severe cases mostly during the first two years of age and rapidly decreases between the second and fourth year of age (Fig A1.2.3 A-B). In the scenario with PMC, up to 600 clinical and 60 severe cases per 1000 population were averted in the first year of life. While the initial number of cases averted was greatest at high transmission intensity, so was the decline in effect even leading to some increase in cases after the intervention stopped that remained for several years for clinical malaria, while quickly resolved for severe malaria (Fig A1.2.3 B). In the scenario with RTS,S the total number of cases averted increased during the first and reached their maximum during the second year of life. Notably, RTS,S was projected to lead to some rebound in clinical malaria that lasted for at least five years, whereas the rebound in severe malaria was very small and quickly resolved (Fig A1.2.3 B).


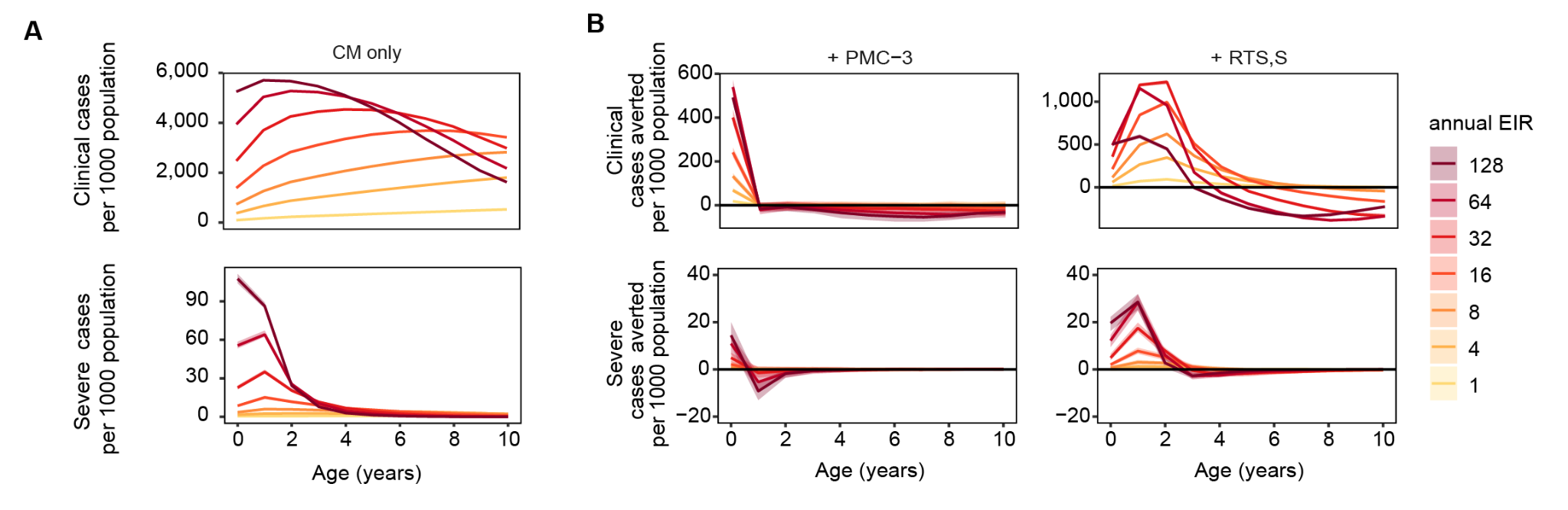


**Fig A1.2.3: Projected clinical and severe malaria cases and cases averted over age in years and by EIR.**

**A)** Clinical and severe malaria cases by age for children U5 without either RTS,S or PMC-3. **B)** Clinical and severe malaria cases by age with PMC-3 or RTS,S at 80% intervention coverage. Arrows indicate the timing of each PMC dose, the 3rd RTS,S priming dose, and the RTS,S booster dose.


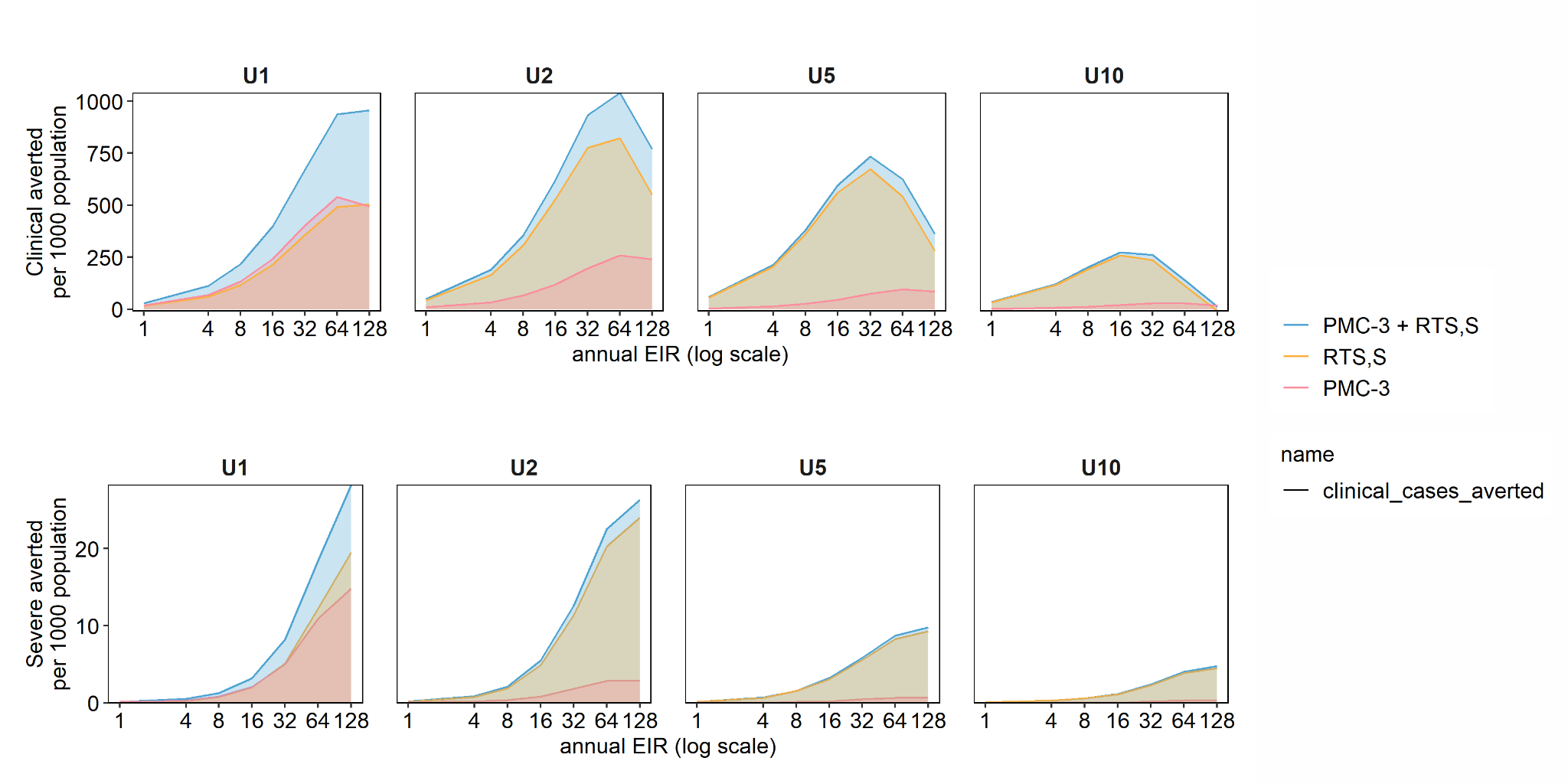


**Fig A1.2.4:** Cases averted per age group and EIR for PMC-3, RTS,S and PMC-3+RTS,S.

The solid line shows the median across stochastic runs and birth-cohorts.

**Impact of PMC + RTS,S by PMC schedule**

Both interventions together nearly depleted new episodes for a short period of time and slowed down the increase in incidence between PMC doses, when assuming 80% coverage for both.

At moderate-high transmission, adding RTS,S averted four to six times (PMC-3) or two to three times (PMC-5a and PMC-7) more cases per 1000 children U2 compared to PMC alone. When PMC was added to RTS,S, an additional 314 (20%) clinical and 2 (8%) severe cases were averted per 1000 children U2 (EIR=32 ibpa). Both interventions together averted fewer cases than the sum of cases averted by each intervention alone. For instance, PMC-3 + RTS,S averted on average 2,016 clinical cases, while PMC-3 averted 501 cases and RTS,S 1,549 cases per 1000 population U2. Of the combined impact, 77% were attributable to RTS,S and 23% were attributable to PMC-3, mostly during the first year of life (Fig A1.2.5A).

When RTS,S was added to PMC (‘PMC first’), an additional 757, 735, and 741 cases per 1000 population U2 were averted for PMC-3, PMC-5, and PMC-7 respectively, corresponding to a 100-300% increase compared to the impact of PMC alone (cases averted by PMC alone: 250, 449, and 668 for PMC-3, PMC-5 and PMC-7 respectively). When PMC was added to RTS,S (‘RTS,S first‘), an additional 233, 410 and 636 cases per 1000 population U2 were averted, by PMC-3, PMC-5, and PMC-7 respectively (cases averted by RTS,S alone: 774 per 1000 population U2 per year) (Fig A1.2.5B).


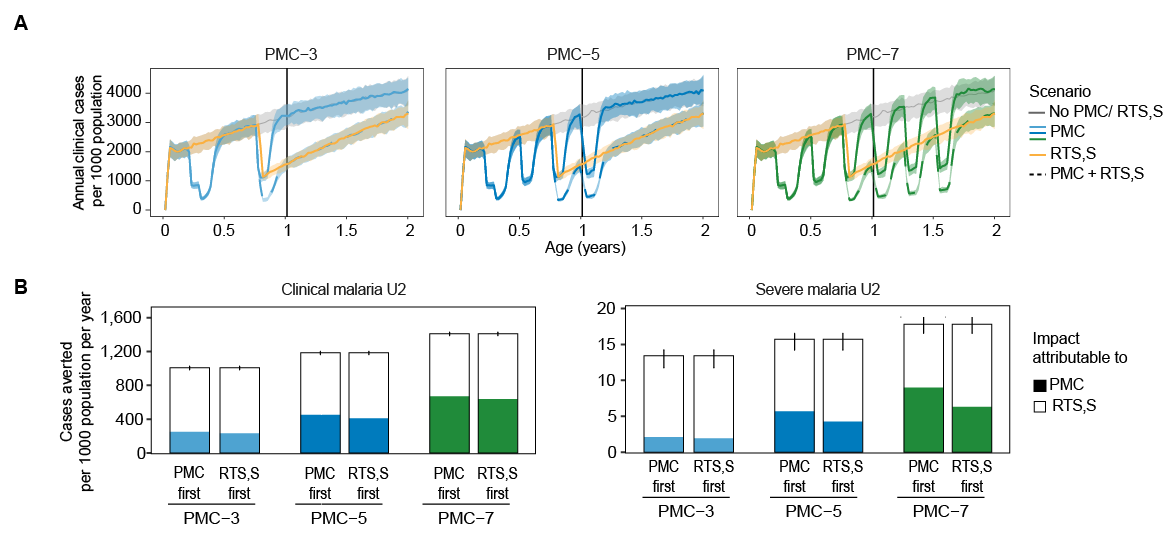


**Fig A1.2.5: Predicted impact on malaria cases of PMC and RTS,S by PMC schedule in children U2.**

**A)** Age-incidence curve for PMC-3, RTS,S and PMC-3+ RTS,S. The solid line shows median and shaded area 90% PI based on model and seasonality uncertainty. The gray line shows the counterfactual and the colored lines PMC or RTS,S. **B)** Cases averted per 1000 population U2 per year attributable to PMC or RTS,S in the scenario where both were deployed. Coverage was set to 80% for both interventions.


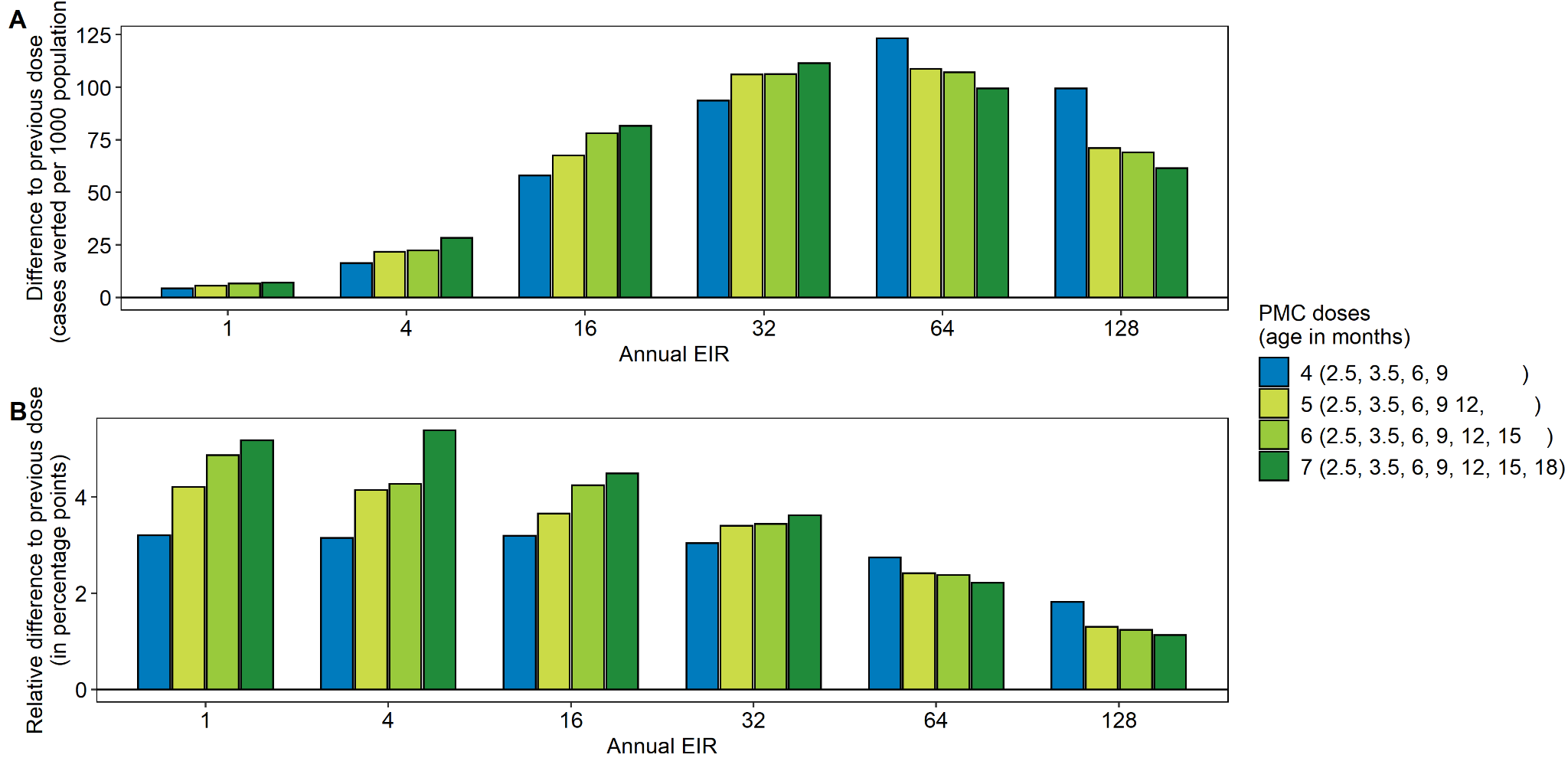


**Fig A1.2.6: Incremental impact of additional PMC doses on clinical cases in children under the age of 2 years by transmission intensity**. A) additional cases averted per 100 population U2. B) Percentage points increase in relative number of cases averted in children U2. Projections based on the geographic-agnostic model run with 80% coverage per dose and effective treatment coverage at 60% for clinical and 80% for severe cases.


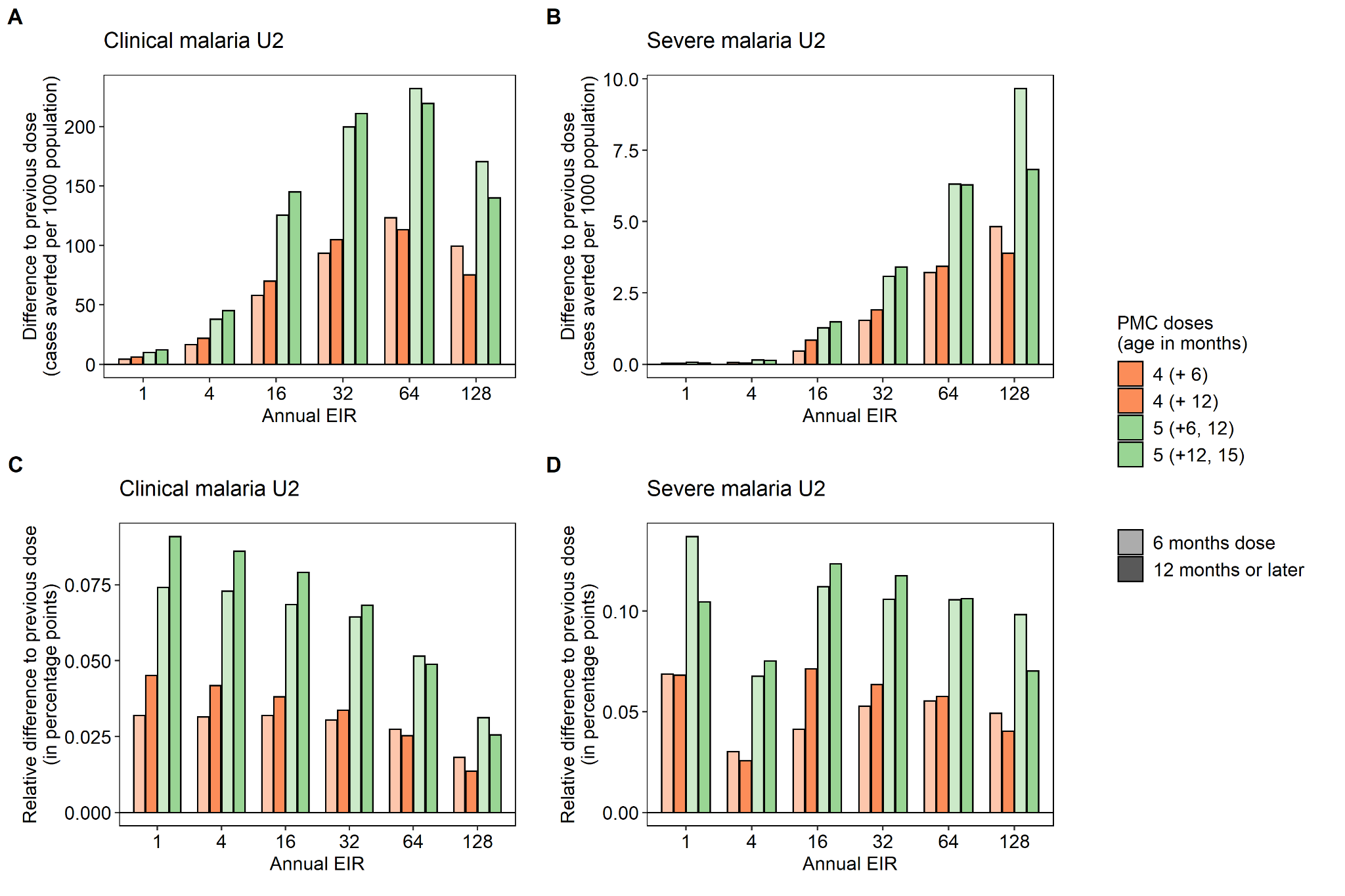


**Fig A1.2.7: Incremental impact of PMC-4 and PMC-5 with doses during infancy or extended into the 2nd year of life in comparison to PMC-3 on clinical cases in children under the age of 2 years by transmission intensity**. **A+C)** additional cases averted per 100 population U2. **B+D)** Percentage points increase in relative number of cases averted in children U2. Projections based on the geographic-agnostic model run with 80% coverage per dose and effective treatment coverage at 60% for clinical and 80% for severe cases.


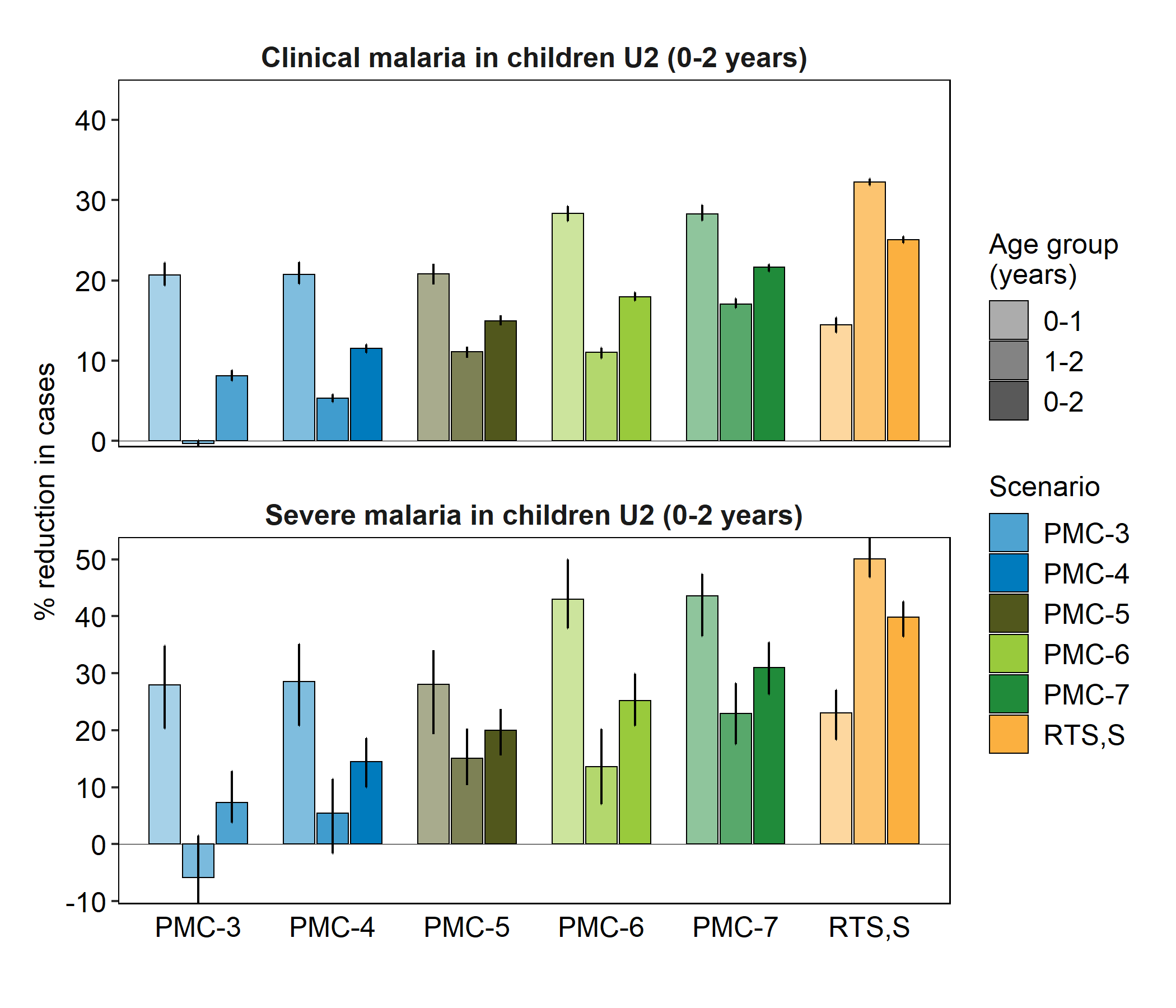


**Fig A1.2.8:** Relative reductions in clinical and severe cases by PMC-RTS,S scenario at 80% coverage for children 0-1 and 1-2 years and U2 (0-2 years). Error bars show 90% PI across birth cohorts and stochastic replications.


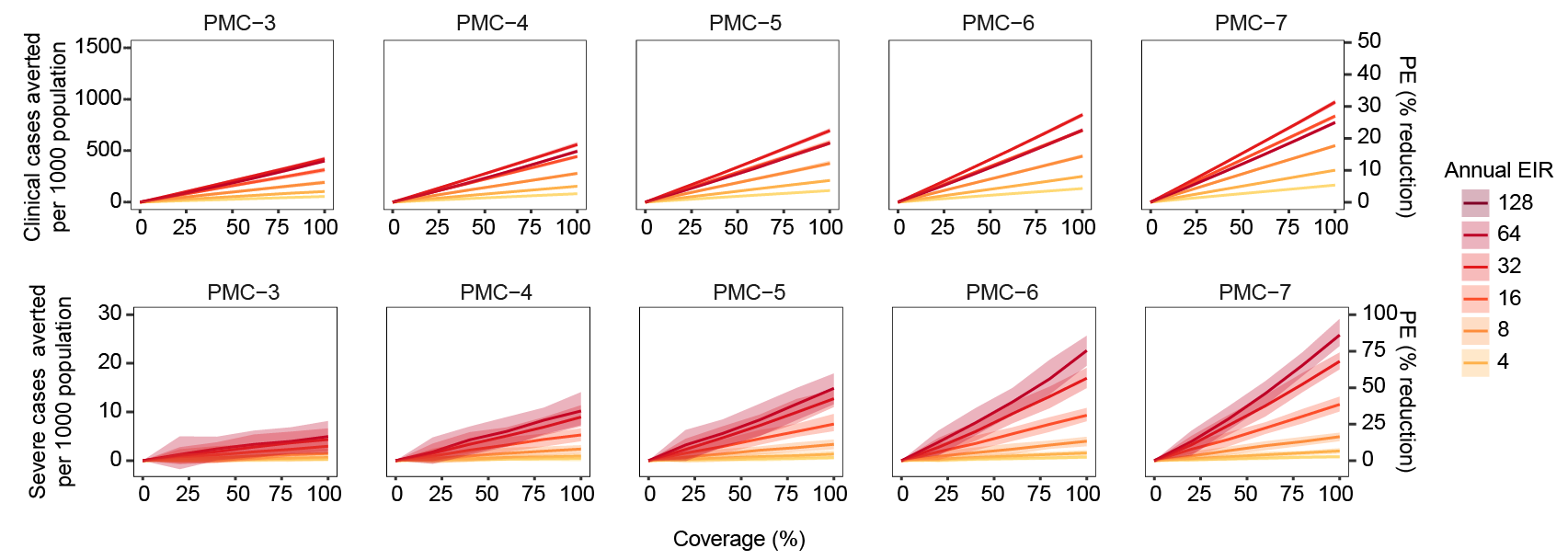


**Fig A1.2.9:** Number of cases averted per 1000 population per year by PMC-RTS,S scenario at varying coverage levels and transmission intensities in children and U2 (0-2 years of age).

*
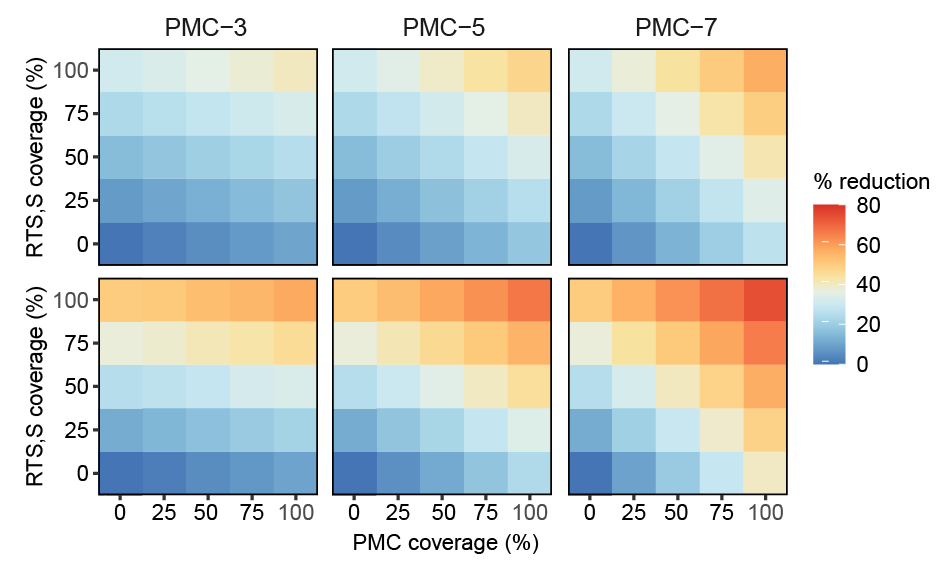
*

**Fig A1.2.10:** Coverage matrix of PMC and RTS,S and projected relative reductions in clinical and severe malaria in children U2. Projections based on the geographic-agnostic model run at transmission intensity of 32 ibppa and effective treatment coverage at 60% for clinical and 80% for severe cases.


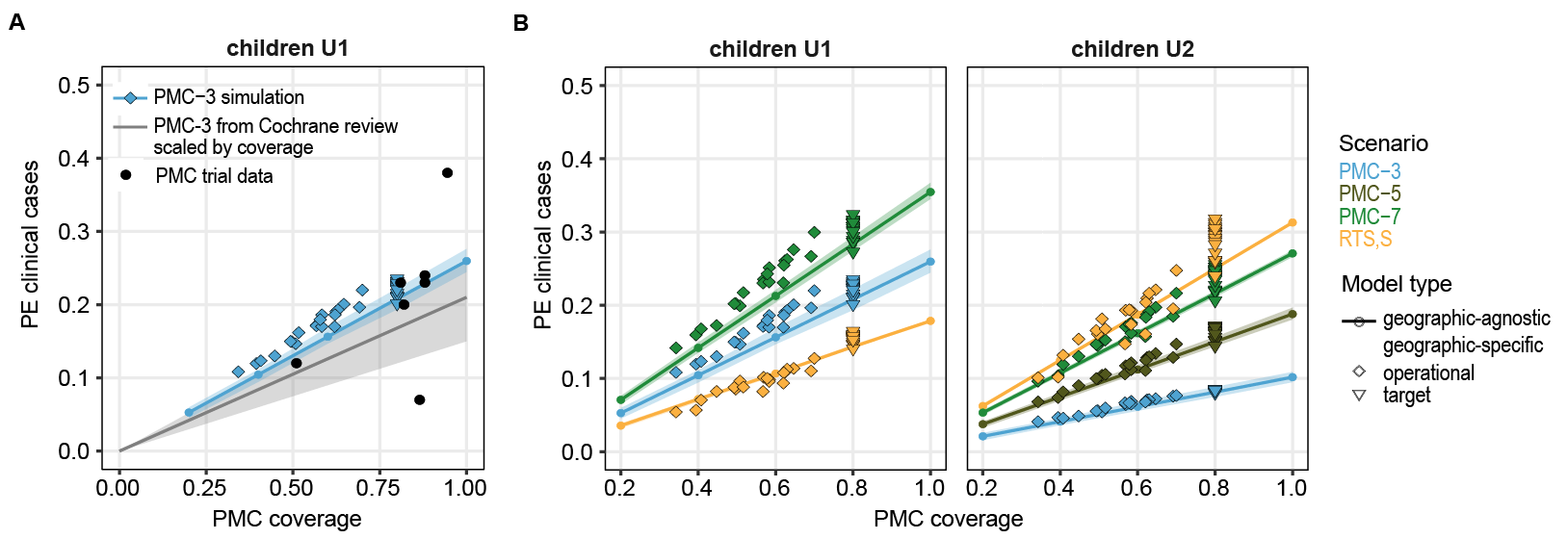


**Fig A1.2.11:** Protective efficacies (PE) by PMC coverage per scenario, model type and age group. **A)** Geographic-agnostic and geographic-specific (see A1.4) model projections for PMC-3 by coverage level in blue. The grey line and shaded area were derived from the PE for clinical malaria estimated in a Cochrane review [3] and the black points show the trial specific PEs included in the review also shown in Fig A1.1.1 B, obtained from [2]. **B)** Geographic-agnostic and geographic-specific (see A1.4) model projections for several PMC scenarios by coverage level in comparison for children under 1 or under 2 years.

## **A1.3: Sensitivity analysis, exploration of parameter uncertainty**

The generic model setup was further used to explore different assumptions on specific parameters that are known or hypothesized to be associated with malaria incidence in infants or directly with the intervention impact. Parameters explored include a) intervention related parameters, such as coverage and timing of PMC doses, as well as correlation between coverage of PMC and RTS,S, or b) biological and context related parameters, such as maternal antibody protection, birth rate seasonality, and case management coverage levels.

### Completeness of PMC doses

In total, three coverage scenarios were explored, a target coverage of 80% randomly distributed, a target coverage of 80% to the same children each dose and an operational coverage scenario with varying coverage per touchpoint informed by EPI coverage (Fig A1.3.1-3). The number of PMC doses received per child varied from 2.4-5.6, 3-7, and 1.9-3.9 doses across PMC doses for each of the coverage scenarios respectively. The two coverage scenarios at 80% resulted in the same impact predictions since the subpopulations of receiving PMC or not distinguished in any other characteristics such as transmission risk or access to case management or other interventions.


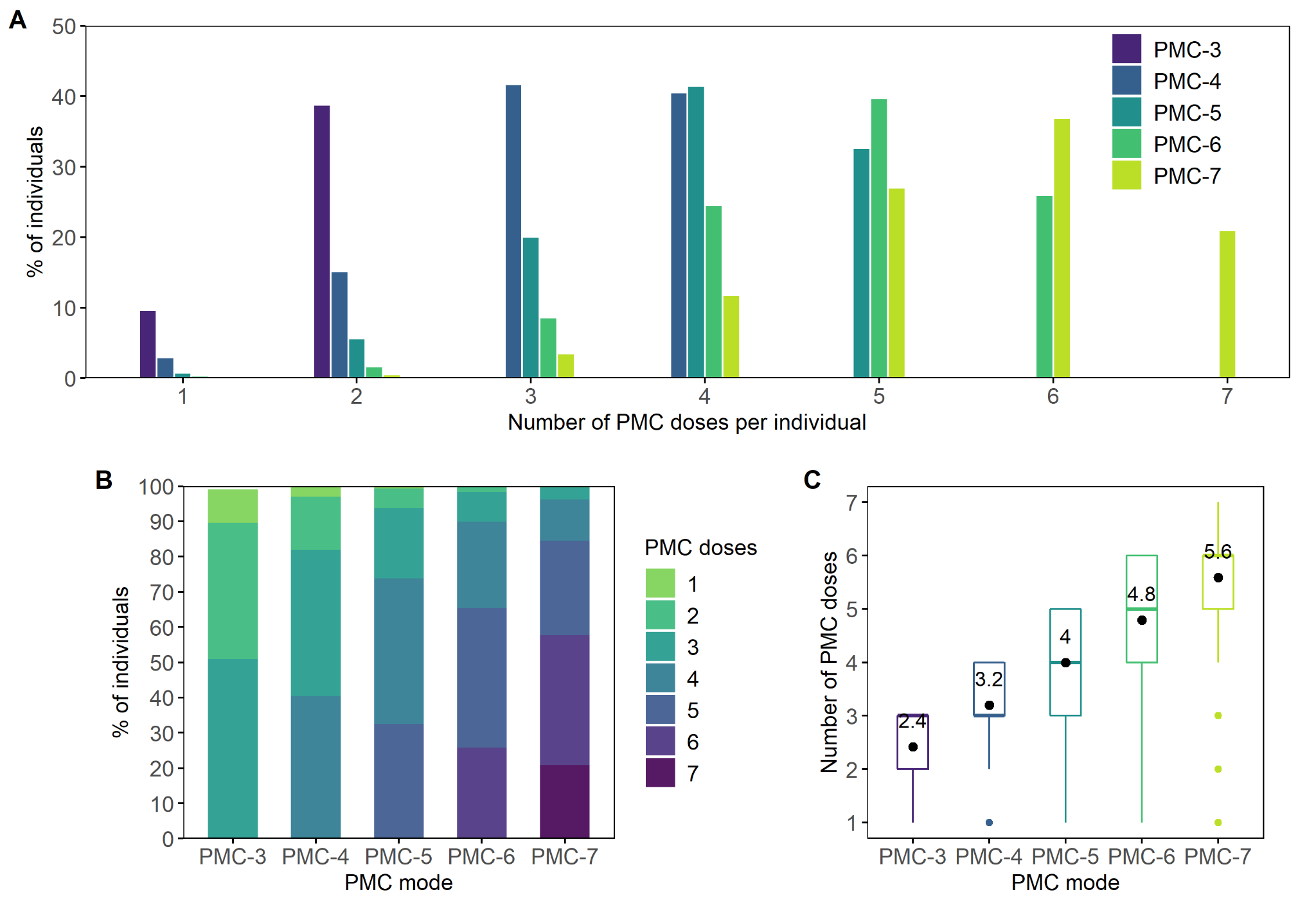


**Fig A1.3.1:** Individual PMC coverage per dose at target coverage (80%).


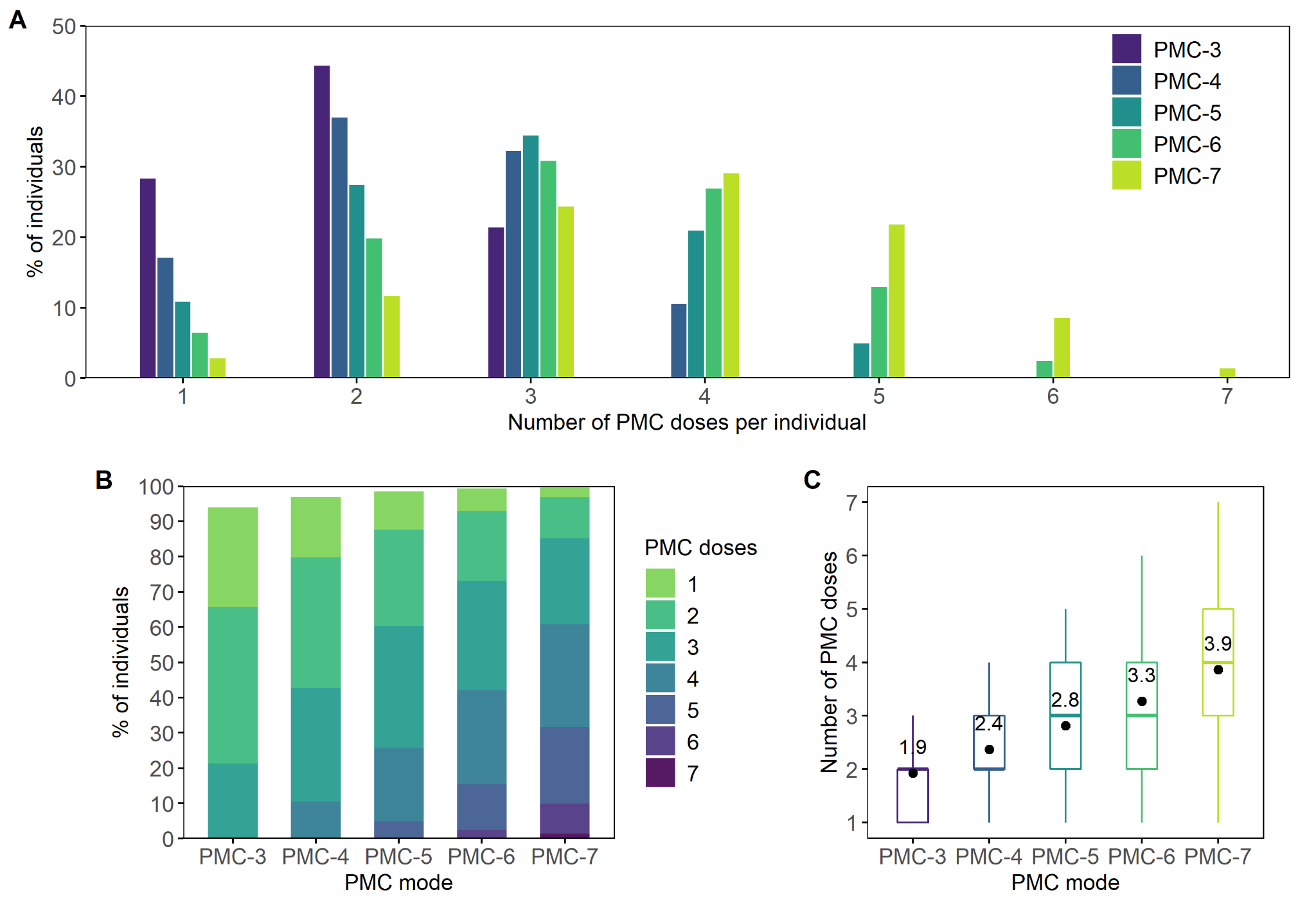


**Fig A1.3.2:** Individual PMC coverage per dose at operational coverage.

**
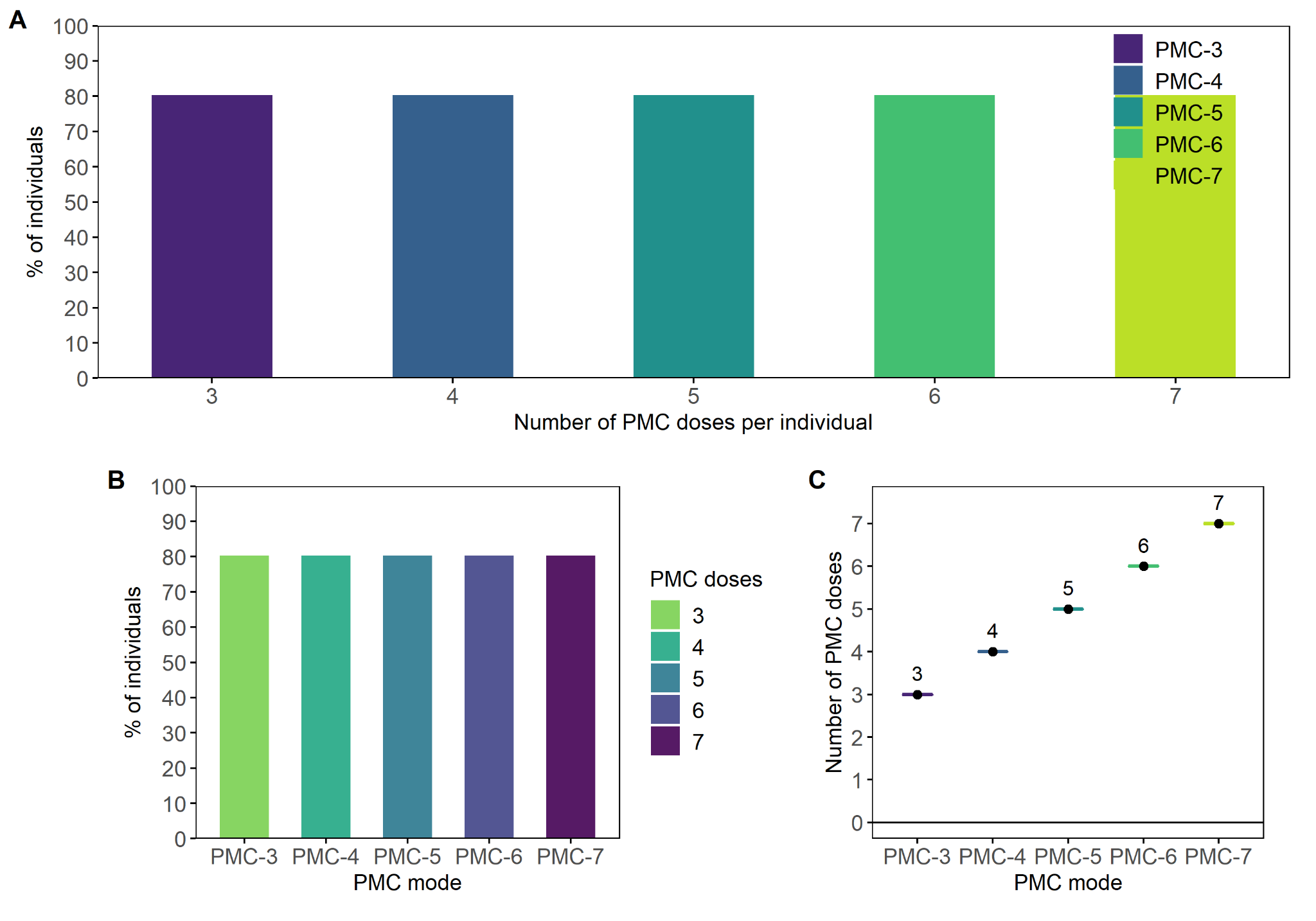
**

**Fig A1.3.3:** Individual PMC coverage per dose at complete 80% coverage.

### Clinical treatment coverage

Additional scenarios were run in which the clinical treatment coverage was varied as well as the PMC coverage, whereas the transmission intensity was held constant at 32 ibpa. PMC was simulated with 3 doses during the first year of life and results presented for children U1. The influence of the clinical treatment coverage differed for clinical and severe malaria with slightly more clinical cases averted by PMC, but fewer severe cases averted by PMC when treatment coverage of clinical cases was high. In EMOD, treating more clinical episodes and clearing more infections allows more individuals to experience a next episode sooner, hence increasing the total number of cases. On a relative scale, the projected relative reduction in cases due to PMC was indistinguishable across the simulated clinical treatment coverage levels (Fig A1.3.2).


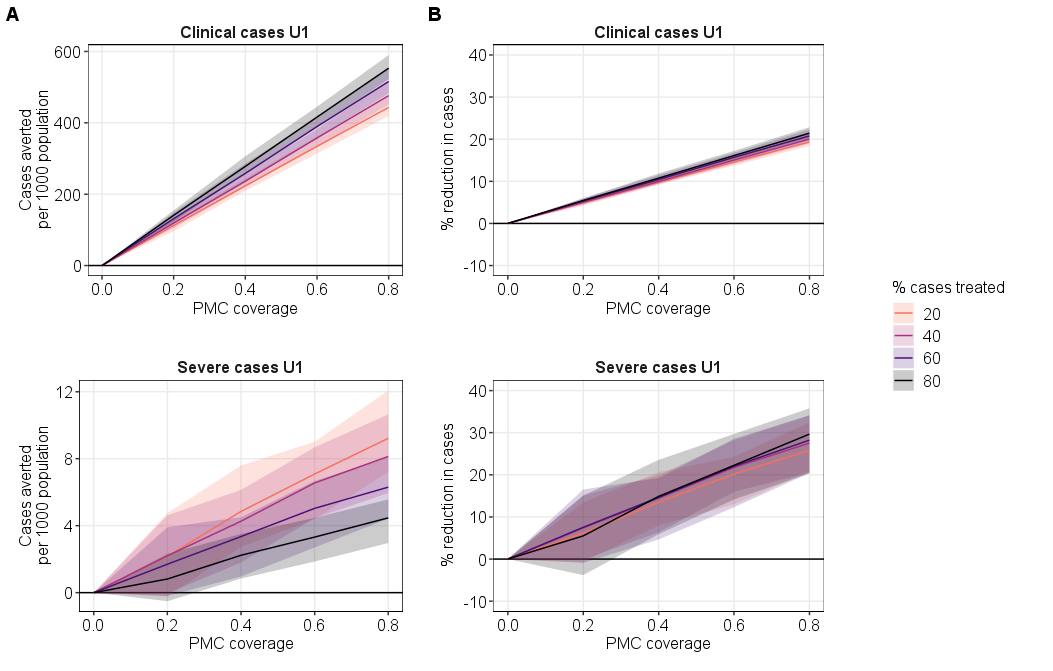


**Fig A1.3.2:** Clinical and severe **c**ases averted (A) and relative reductions (B) in children U1 for PMC-3 at case management coverage intervals of 20%. Simulations ran for a transmission intensity of EIR = 32 ibpa.

### Age-varying clinical treatment coverage

In the model, the same treatment coverage was assumed for children under the age of 5 years. In practice infants are more likely to be taken to a health facility than older children. For instance a study in Sierra Leone found a drop in case management by 7% and by 13% in children aged 12-35 or 36-59 months respectively compared to case management in children <12 months of age [[12]](https://www.zotero.org/google-docs/?vhRhpO). To explore the influence of age-varying case management rate on the simulation results, additional scenarios were run including this variation. Compared to homogeneous treatment coverage, a decrease in treatment coverage by age resulted in more clinical but fewer severe episodes (Fig A1.3.3).


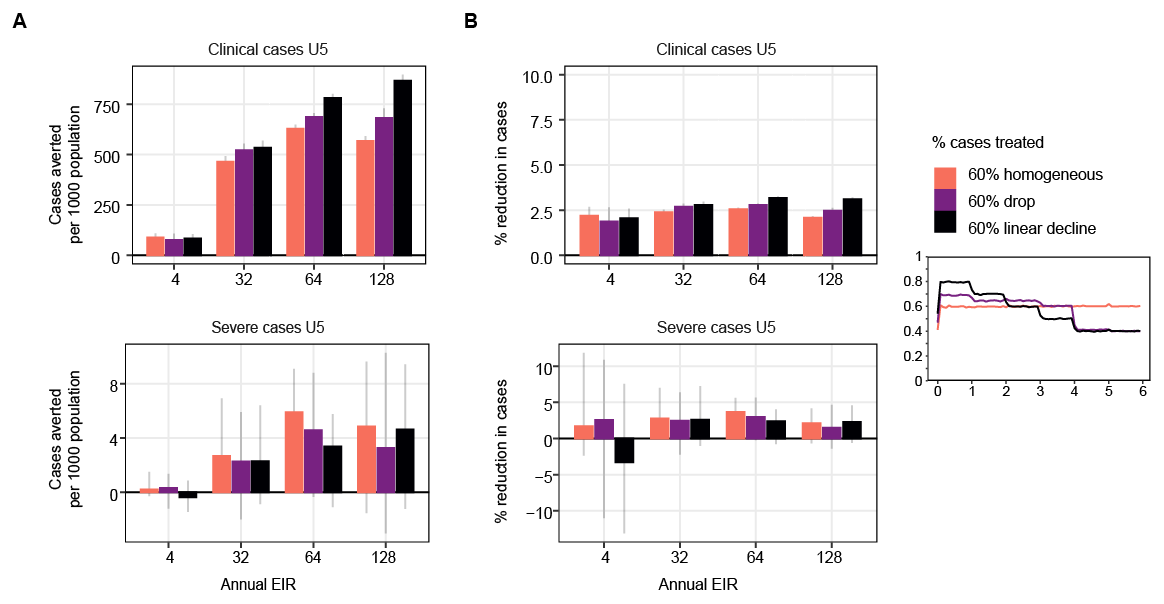


**Fig A1.3.3:** Clinical and severe **c**ases averted (A) and relative reductions (B) in children U5 for PMC-3 at varying case management assumptions. PMC simulated with 80% coverage and at a transmission intensity of EIR = 32 ibpa. The small inset figure shows the pattern of treated cases by age in years on the x-axis.

### Maternal antibody protection

Infants inherit maternal antibodies from their mothers that provide to some extent protection against several pathogens during the first months of life. For malaria, immunity wanes during the first 6 months of life, whereas its mechanisms and effect on infection and disease protection are still not completely understood [REF]. In EMOD maternal antibody protection against malaria in infants is simulated by transferring malaria antibodies from the mother to the newborn. The additional antibodies in the infant result in higher parasite killing rates. The maternal antibodies decay over time with a half-life of three months. The maternal protection is adjusted depending on EIR and then downscaled to account for ‘maternal immunity levels’ [[13]](https://www.zotero.org/google-docs/?QLhThR) (Fig A1.3.4). A maximum difference of 10% in predicted efficacy of PMC for clinical and 20% for severe cases was predicted across the range of simulated strengths of maternal antibody protection in EMOD. The influence of transmission intensity on the variation in relative impact of IPTi depending on strengths of maternal antibody protection was higher for clinical cases than for severe cases (Fig A1.3.5-6).


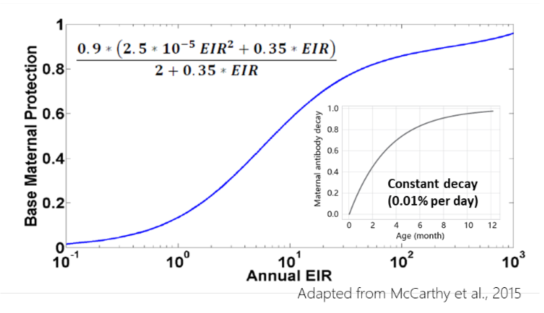

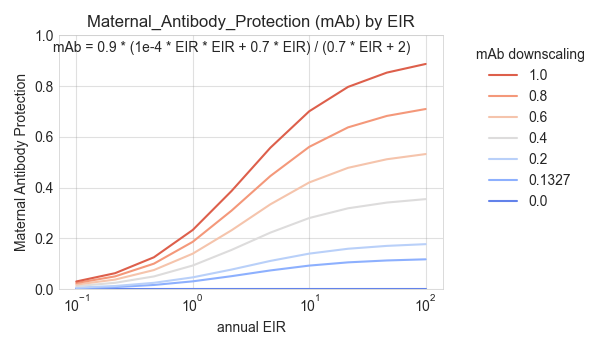


**Fig A1.3.4:** A) Maternal antibody protection and EIR relationship in EMOD, adapted from [13]. B) Adjusted maternal antibody protection for the level of antibodies in mothers.


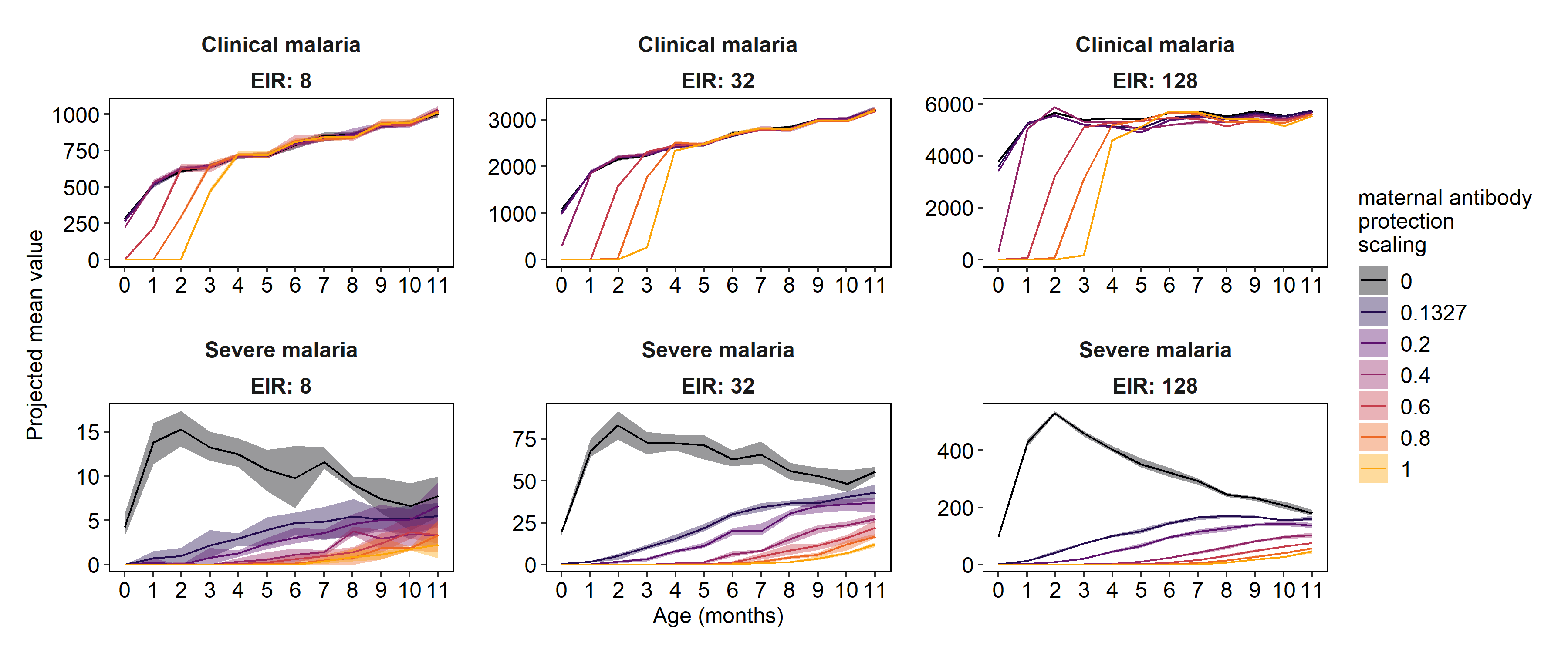


**Fig A1.3.5:** Projected prevalence, clinical and severe incidence in children 0-12 months of age by maternal antibody protection scaling factor. Simulations ran for a single birth cohort (born January) and season1 pattern. Shaded areas show stochastic uncertainty based on 5 seeds.


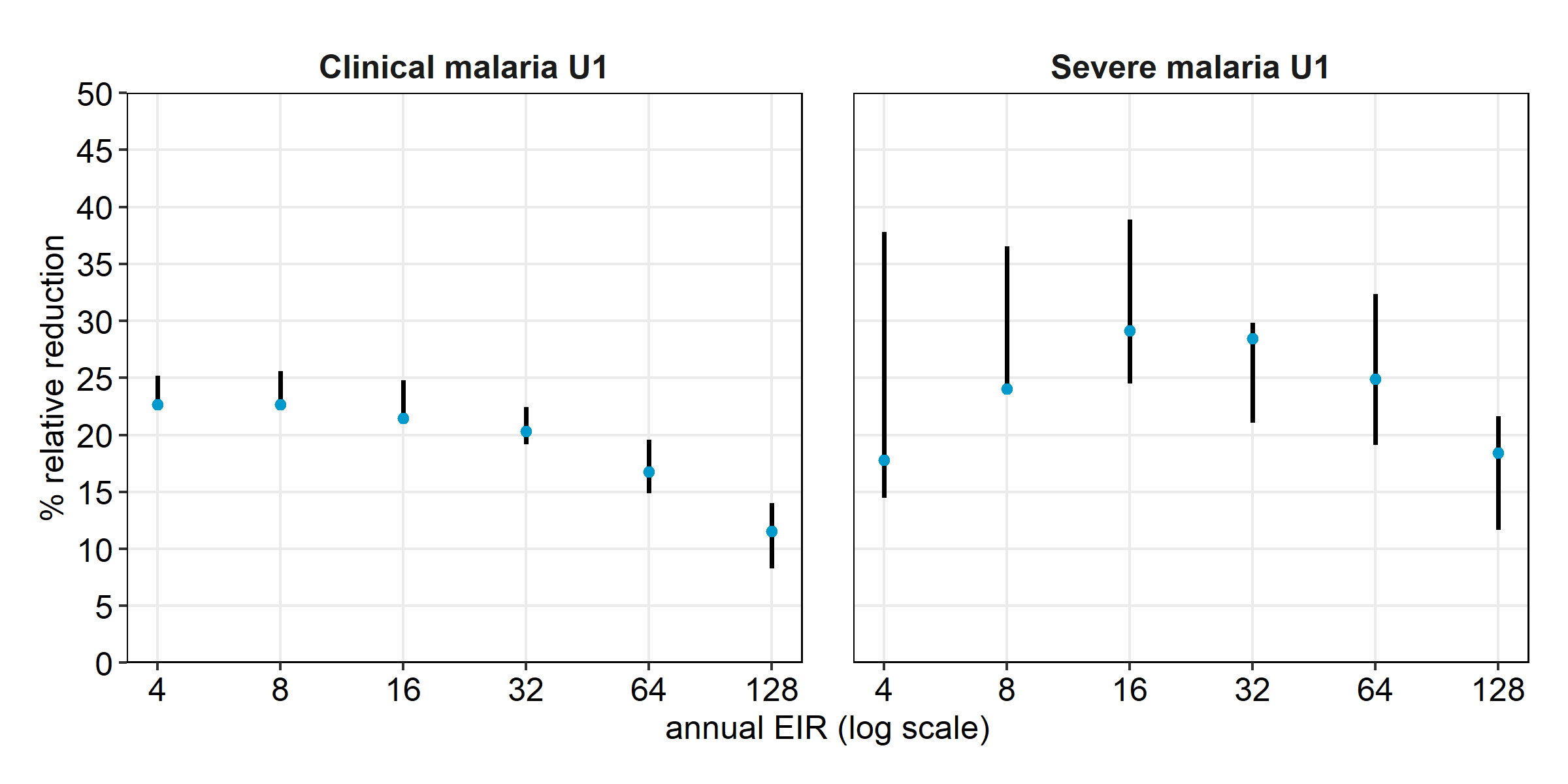

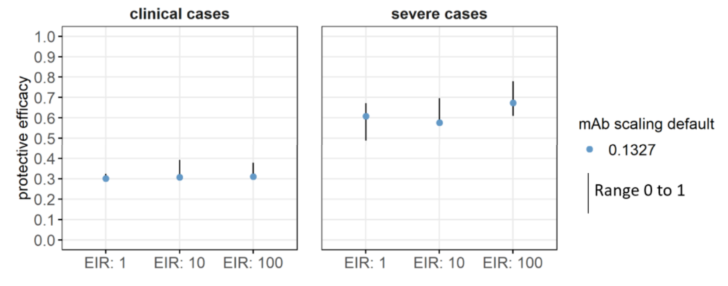


**Fig A1.3.6:** Modeled protective efficacy of PMC-3 on clinical and severe cases in children 0-12 months by EIR. Error bars based on the range of maternal antibody protection scaling.

##

## **A1.4: Country application to Southern Nigeria**

*Population estimates*

Population estimates for all ages were obtained from GeoPode Version 2 for 2019 [[14]](https://www.zotero.org/google-docs/?AqZbIK). Since no disaggregated population data per year for children under the age of five years was available, the simulated population was used to derive the proportion of children under two years of age of ~6.8%.


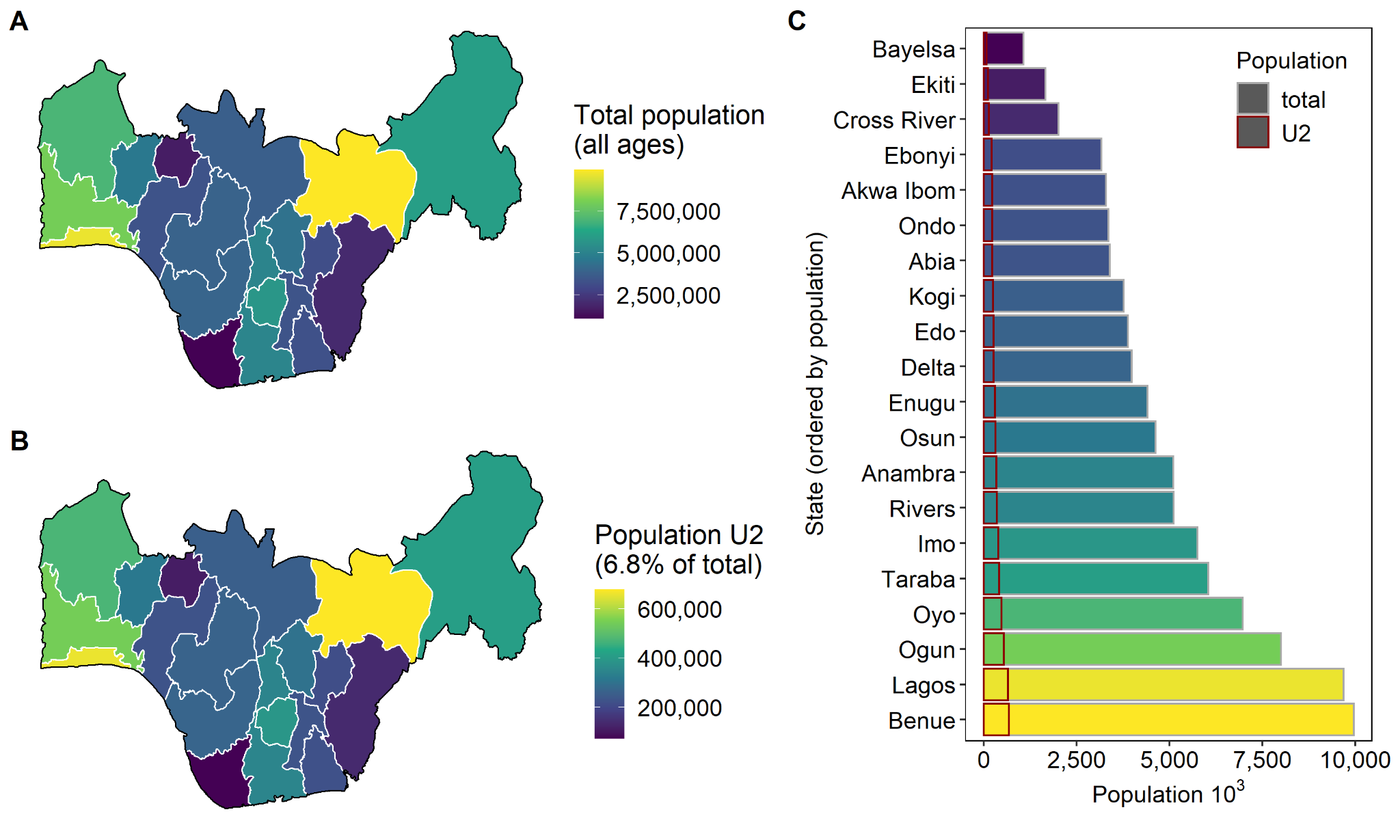


**Fig A1.4.1:** Total population and estimated population U2 in Southern Nigeria for 2019.

*EPI coverage extraction and adjustment*

EPI coverage estimates for DTP-2, DTP-3, and measles per State (admin 1 unit) were obtained from the NDHS 2018 [15], accessing the API via the *rdhs* package [16]. The coverage was adjusted to indirectly account for operational intervention implementation factors, using observed differences in coverage between EPI and PMC in an implementation pilot study in Sierra Leone in 2019 [[17]](https://www.zotero.org/google-docs/?Bc6IUl). In that study, researchers observed lower coverage for IPTi/PMC compared to the EPI vaccines administered at the same age, and their ratio was used to downscale the EPI coverage levels for Nigeria as proxy for PMC coverage.

*Clinical and severe treatment coverage*

The proportion of febrile children under the age of five years who took an artemisinin-based combination therapy in the two weeks preceding the survey from the NDHS 2018 was used as an indicator for effective treatment coverage per clinical case under the age of five years in the simulation. The calculation was performed using household cluster level data in alignment to previous Nigeria modeling analysis [11]. The severe treatment coverage was fixed at an optimistic 80%, as in the geographic-agnostic run.


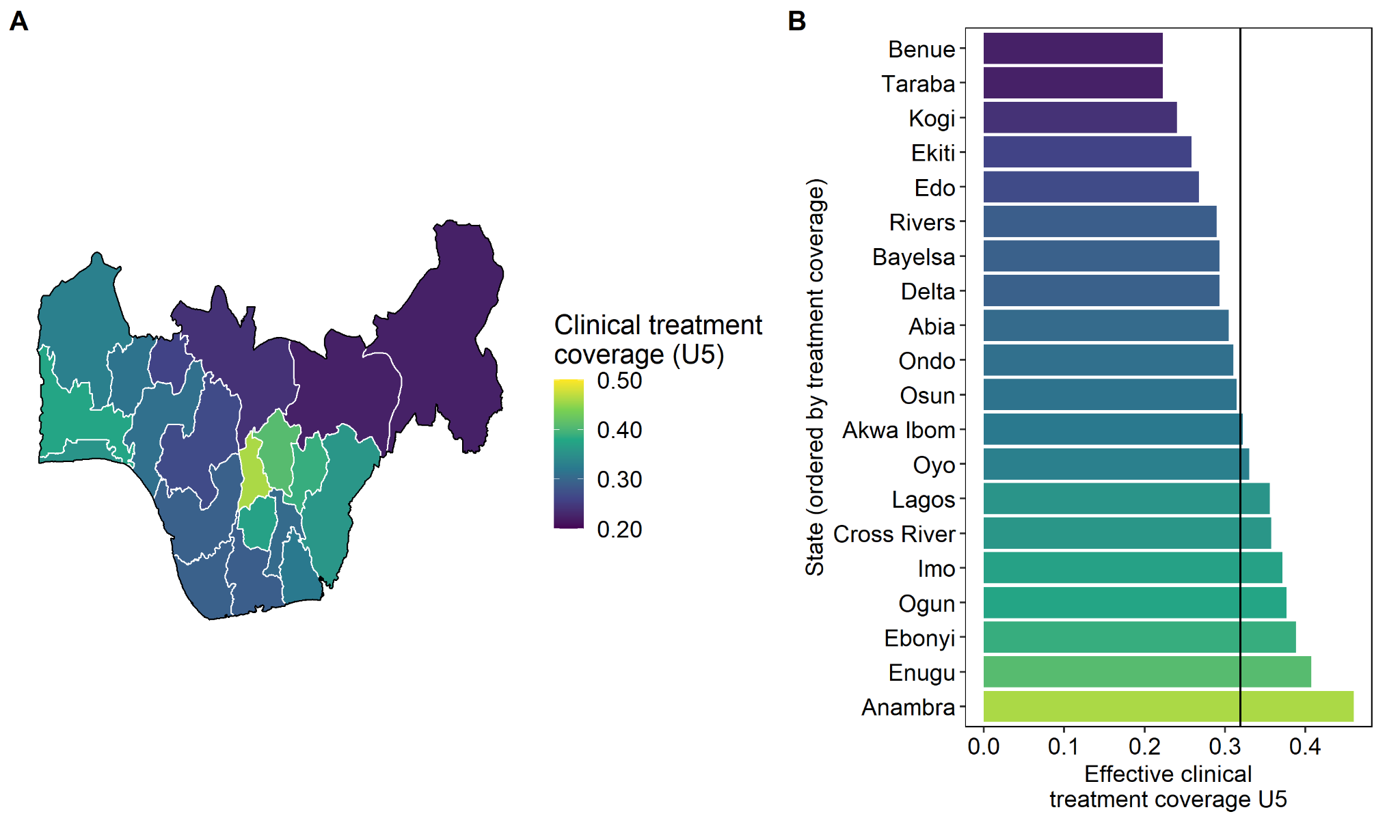


**Fig A1.4.2: A)** Map of clinical treatment coverage in Southern Nigeria and **B)** values per State. Data sources: NDHS 2018 [15].

*Transmission intensity and seasonality*

We extracted State-level malaria prevalence in children U5 (*Pf*PR_0.25-5_) based on rapid diagnostic tests, EPI coverage in children 12-23 months of age, and case management coverage in children U5 from the NDHS 2018. To obtain appropriate input EIR level for each State, we used the *Pf*PR_0.25-5_ -EIR relationship from previous simulation runs (Fig A1.4.3 A-B).

To inform transmission seasonality, monthly mean EIR values for 2020 per State were obtained from the simulation outputs from a previous EMOD model calibrated to monthly incidence and survey malaria prevalence in Nigeria [[11]](https://www.zotero.org/google-docs/?PRViO9) (Fig A1.4.3 C).

*
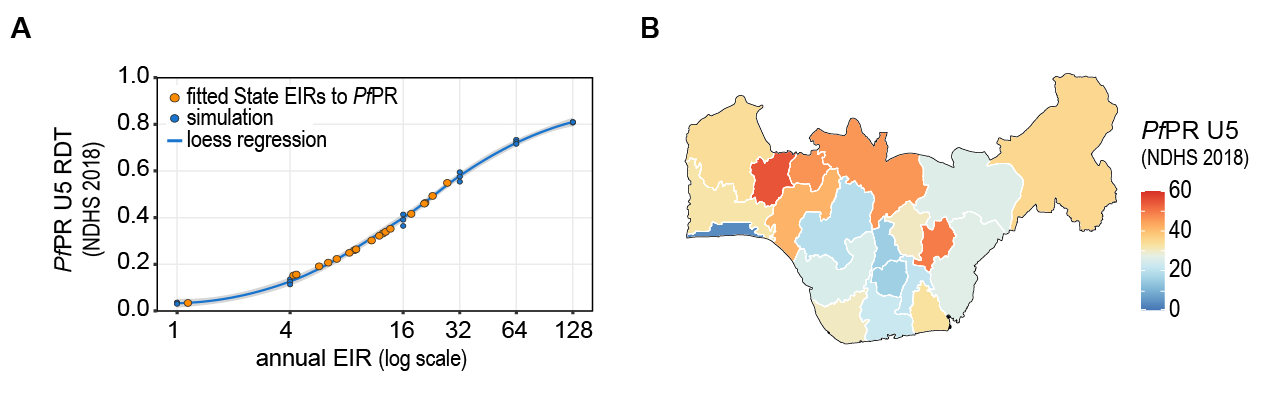
*


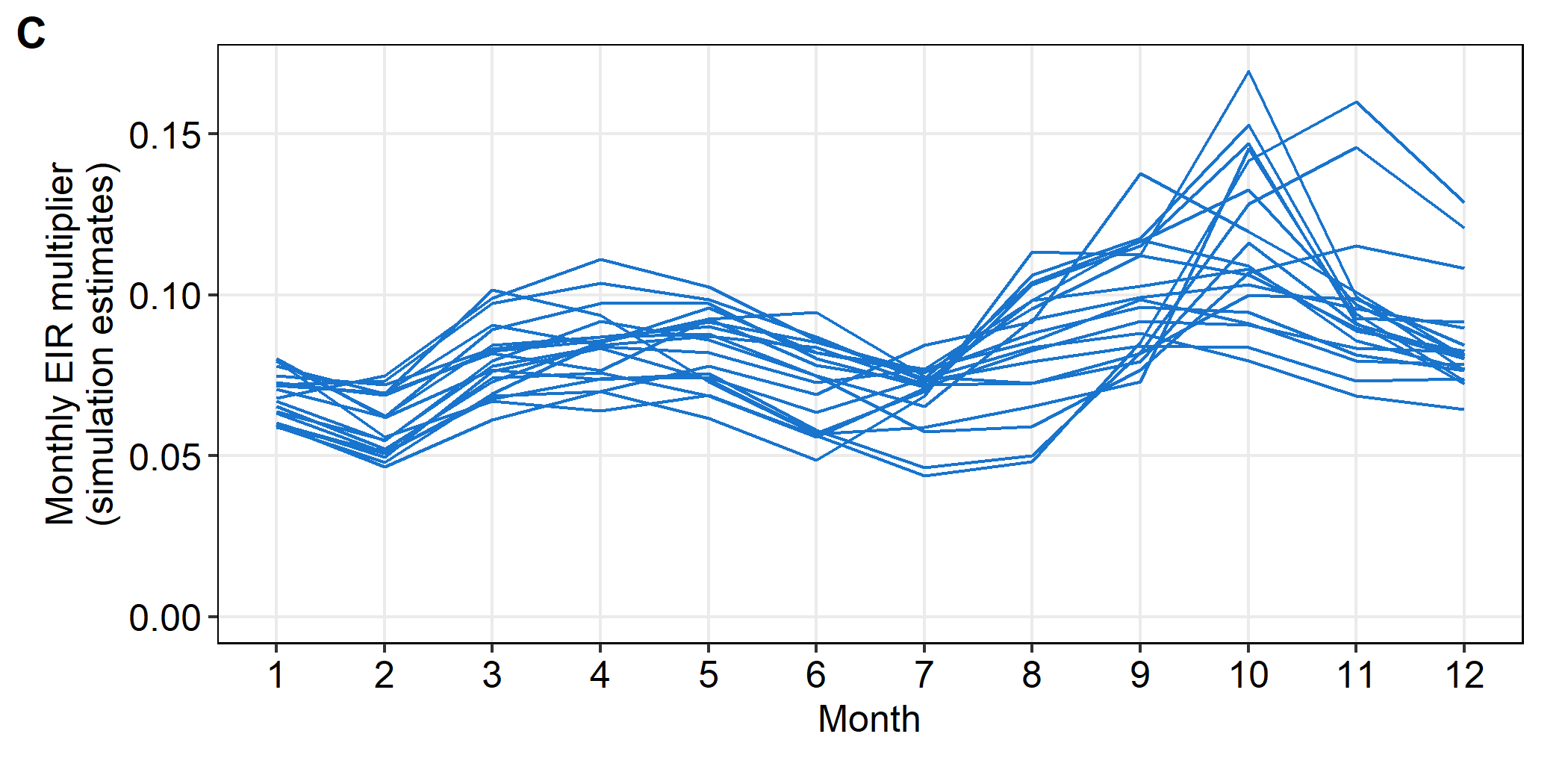


**Fig A1.4.3: A)** Simulated EIR to prevalence relationship used to match DHS prevalence to simulation estimated EIR levels per State. **B)** Map of malaria prevalence in children under the age of five years based on RDT. **C).** Transmission seasonality multipliers per State.

*Additional result figures*

Clinical and severe cases averted per 1000 population in Southern Nigeria.


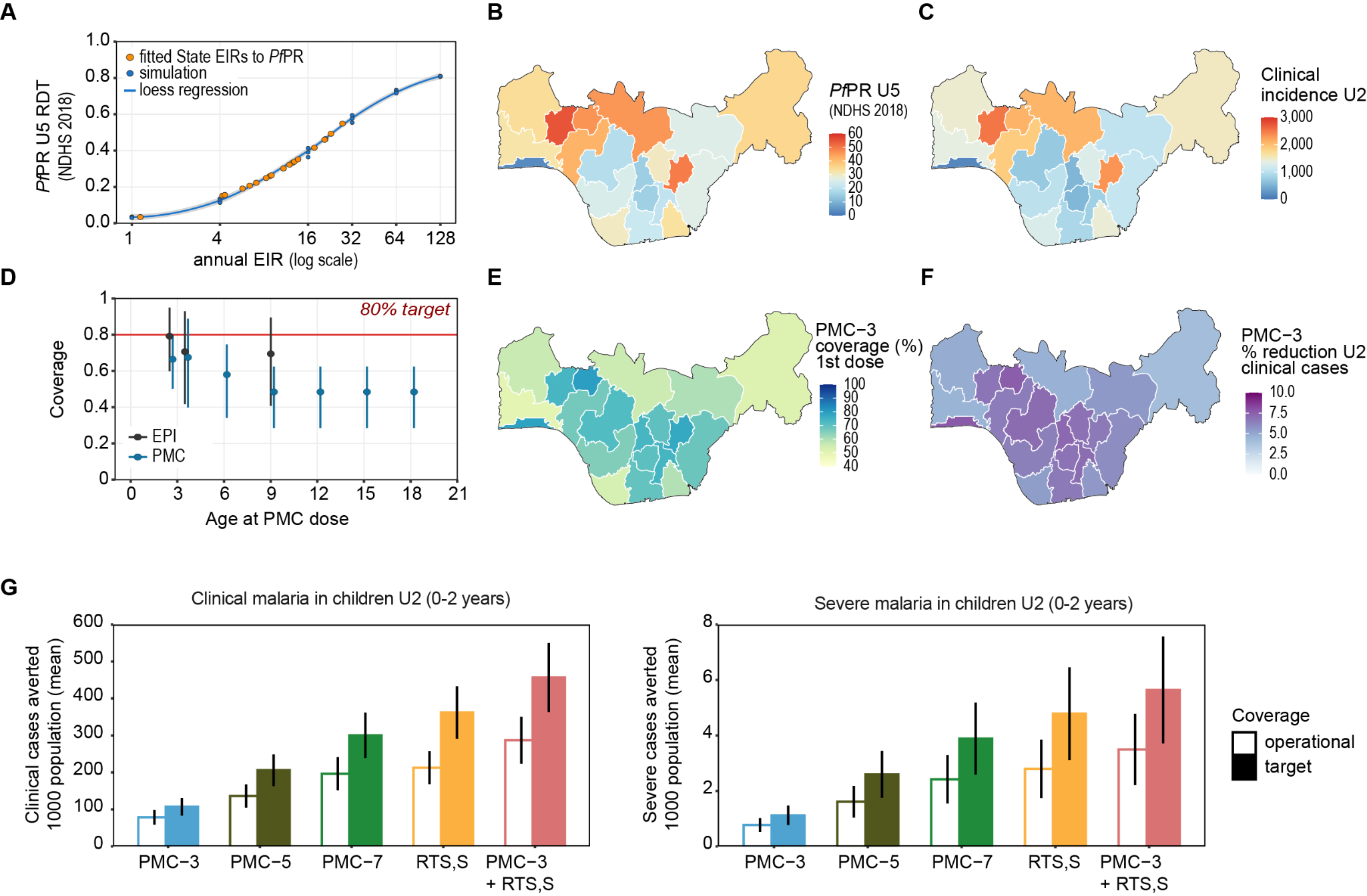


**Fig A1.4.4:** Projected intervention impact at operational and target coverage for Southern Nigeria, described in annual cases averted per 1000 population.

## **References**

[1. Cairns M, Carneiro I, Milligan P, Owusu-Agyei S, Awine T, Gosling R, et al. Duration of Protection against Malaria and Anaemia Provided by Intermittent Preventive Treatment in Infants in Navrongo, Ghana. PLOS ONE. 2008;3: e2227. doi:10.1371/journal.pone.0002227](https://www.zotero.org/google-docs/?yEqeGj)

[2. Aponte JJ, Schellenberg D, Egan A, Breckenridge A, Carneiro I, Critchley J, et al. Efficacy and safety of intermittent preventive treatment with sulfadoxine-pyrimethamine for malaria in African infants: a pooled analysis of six randomised, placebo-controlled trials. The Lancet. 2009;374: 1533–1542. doi:10.1016/S0140-6736(09)61258-7](https://www.zotero.org/google-docs/?yEqeGj)

[3. Esu EB, Oringanje C, Meremikwu MM. Intermittent preventive treatment for malaria in infants. Cochrane Database Syst Rev. 2021 [cited 22 Jul 2021]. doi:10.1002/14651858.CD011525.pub3](https://www.zotero.org/google-docs/?yEqeGj)

[4. Chandramohan D, Owusu-Agyei S, Carneiro I, Awine T, Amponsa-Achiano K, Mensah N, et al. Cluster randomised trial of intermittent preventive treatment for malaria in infants in area of high, seasonal transmission in Ghana. BMJ. 2005;331: 727–733.](https://www.zotero.org/google-docs/?yEqeGj)

[5. Ridley RG. Medical need, scientific opportunity and the drive for antimalarial drugs. Nature. 2002;415: 686–693. doi:10.1038/415686a](https://www.zotero.org/google-docs/?yEqeGj)

[6. Sidhu ABS, Verdier-Pinard D, Fidock DA. Chloroquine Resistance in Plasmodium falciparum Malaria Parasites Conferred by pfcrt Mutations. Science. 2002;298: 210–213. doi:10.1126/science.1074045](https://www.zotero.org/google-docs/?yEqeGj)

[7. Owusu-Agyei S, Awini E, Anto F, Mensah-Afful T, Adjuik M, Hodgson A, et al. Assessing malaria control in the Kassena-Nankana district of northern Ghana through repeated surveys using the RBM tools. Malar J. 2007;6: 103. doi:10.1186/1475-2875-6-103](https://www.zotero.org/google-docs/?yEqeGj)

[8. Kasasa S, Asoala V, Gosoniu L, Anto F, Adjuik M, Tindana C, et al. Spatio-temporal malaria transmission patterns in Navrongo demographic surveillance site, northern Ghana. Malar J. 2013;12: 63. doi:10.1186/1475-2875-12-63](https://www.zotero.org/google-docs/?yEqeGj)

[9. RTS,S Clinical Trials Partnership. Efficacy and safety of RTS,S/AS01 malaria vaccine with or without a booster dose in infants and children in Africa: final results of a phase 3, individually randomised, controlled trial. Lancet Lond Engl. 2015;386: 31–45. doi:10.1016/S0140-6736(15)60721-8](https://www.zotero.org/google-docs/?yEqeGj)

[10. Penny MA, Verity R, Bever CA, Sauboin C, Galactionova K, Flasche S, et al. Public health impact and cost-effectiveness of the RTS,S/AS01 malaria vaccine: a systematic comparison of predictions from four mathematical models. The Lancet. 2016;387: 367–375. doi:10.1016/S0140-6736(15)00725-4](https://www.zotero.org/google-docs/?yEqeGj)

[11. Ozodiegwu ID, Ambrose M, Galatas B, Runge M, Nandi A, Okuneye K, et al. Application of mathematical modeling to inform national malaria intervention planning in Nigeria. Research Square. 2022;PREPRINT. doi:10.21203/rs.3.rs-2335288/v1](https://www.zotero.org/google-docs/?yEqeGj)

[12. Bognini JD, Samadoulougou S, Ouedraogo M, Smart F, Kankoye DT, Sankoh O, et al. What are the trends in seeking health care for fever in children under-five in Sierra Leone? evidence from four population-based studies before and after the free health care initiative. PLOS ONE. 2022;17: e0263364. doi:10.1371/journal.pone.0263364](https://www.zotero.org/google-docs/?yEqeGj)

[13. McCarthy KA, Wenger EA, Huynh GH, Eckhoff PA. Calibration of an intrahost malaria model and parameter ensemble evaluation of a pre-erythrocytic vaccine. Malar J. 2015;14: 6. doi:10.1186/1475-2875-14-6](https://www.zotero.org/google-docs/?yEqeGj)

[14. WorldPop and National Population Commission of Nigeria. Bottom-up gridded population estimates for Nigeria, version 2.0. 17 Nov 2021 [cited 1 Dec 2022]. doi:10.5258/SOTON/WP00729.](https://www.zotero.org/google-docs/?yEqeGj)

15. National Population Commission (NPC) [Nigeria] and ICF. 2019. Nigeria Demographic and Health Survey 2018. Abuja, Nigeria, and Rockville, Maryland, USA: NPC and ICF.

16. Watson OJ, FitzJohn R, Eaton JW (2019). “rdhs: an R package to interact with The Demographic and Health Surveys (DHS) Program datasets.” Wellcome Open Research, 4, 103. doi:10.12688/wellcomeopenres.15311.1, https://wellcomeopenresearch.org/articles/4-103/v1.

[17. Lahuerta M, Sutton R, Mansaray A, Eleeza O, Gleason B, Akinjeji A, et al. Evaluation of health system readiness and coverage of intermittent preventive treatment of malaria in infants (IPTi) in Kambia district to inform national scale-up in Sierra Leone. Malar J. 2021;20: 74. doi:10.1186/s12936-021-03615-3](https://www.zotero.org/google-docs/?yEqeGj)

1. [https://docs.idmod.org/projects/emod-malaria/en/latest/parameter-campaign-waningeffects.html](https://docs.idmod.org/projects/emod-malaria/en/latest/parameter-campaign-waningeffects.html#waningeffectboxexponential) [↑](#footnote-ref-1)
